# Youths’ sensitivity to social media feedback: A computational account

**DOI:** 10.1126/sciadv.adp8775

**Published:** 2024-10-23

**Authors:** Ana da Silva Pinho, Violeta Céspedes Izquierdo, Björn Lindström, Wouter van den Bos

**Affiliations:** ^1^ Department of Psychology, University of Amsterdam, Amsterdam, Netherlands.; ^2^ Department of Clinical Neuroscience, Karolinska Institutet, Stockholm, Sweden.; ^3^ Center for Adaptive Rationality, Max Planck Institute for Human Development, Berlin, Germany.

## Abstract

While it is often argued that continuous exposure to social feedback is specifically challenging for the hypersensitive developing brain, empirical evidence is lacking. Across three studies, we reveal the developmental differences and computational mechanisms that underlie the social media engagement and feedback processing of adolescents and adults. First, using a reinforcement learning model on a large Instagram trace dataset (*N* = 16,613, 1.6+ million posts), we show that adolescents are more sensitive to social feedback than adults. Second, in an experimental study (*N* = 194), we show that adolescents’ mood is affected more strongly by a reduction in likes than adults. Last, in a neuroimaging study (*N* = 96), we show that social media feedback sensitivity is related to individual differences in subcortical-limbic brain volumes of emerging adults. Together, these findings highlight the need for digital competence programs to help youth manage the constant feedback they encounter on social media platforms.

## Teaser

Adolescents are more sensitive to social media feedback than adults, and their mood is affected by variations in likes.

## INTRODUCTION

Present-day youth is growing up in a social media–saturated world where technology plays a central role in shaping most of their experiences. Access to social media has become indispensable in the lives of today’s youth, commonly defined as individuals aged 15 to 24 (*1*). Here, we focus on two developmentally distinct yet partly overlapping periods within this category: adolescence (studies 1 and 2; included ages: 13 to 20) and emerging adulthood (study 3; included ages: 18 to 24). These developmental stages integrate distinct biological, social, and psychological changes. Adolescence is marked by puberty, physical changes, identity exploration, increased independence, and the development of more complex cognitive abilities (*2*). Emerging adulthood not only involves further psychosocial development, including identity formation, but also transitions to higher education or the job market, as well as financial independence (*3*). Hence, social media may affect individuals differently depending on the developmental window. The rise of social media use created parental and societal fears over youth’s social and psychological well-being (*4*, *5*), suggesting that the impact of social media on the still-maturing brain increases the chances of developing addictive behaviors or depressive symptoms (*6*–*8*).

One of the main worries is that youths are repeatedly driven to engage in social media use by their increased sensitivity to social feedback and a strong need to belong (*6*, *9*, *10*). Receiving likes on social media is experienced as socially rewarding by recipients (*11*, *12*), results in temporary increases in self-esteem (*13*), and is reported to provide youth with a sense of belonging (*14*). However, likes have also been shown to be strong reinforcers driving social media engagement in adults, which may lead to compulsive or addiction-like behaviors (*11*, *15*, *16*). Furthermore, not receiving feedback can be experienced as social rejection and can reduce self-esteem (*17*, *18*). Adolescence is a developmental period during which both reward and rejection sensitivity are particularly strong (*19*, *20*) and have, respectively, been linked to increased impulsive behavior (*21*, *22*) and depressive symptoms (*23*, *24*). Together, these results support the hypothesis that social media feedback may play a direct role in both increased social media engagement and mental health outcomes for youth. However, there are currently no studies that have directly investigated how youths respond to social feedback on social media platforms.

Over the past decade, research on the link between social media use and mental health outcomes has not yielded consistent results (*25*, *26*). Recent meta-analyses and reviews have identified an overreliance on subjective and high-level measures of social media use such as self-reported screen time, as a key limitation in the field (*27*, *28*). Screen time reveals little about what youths experience or what they do online, and recently, it has been shown that self-reported screen time is not even a good predictor of objectively recorded screen time (*29*). Considering the widespread mental health crisis among young people (*30*, *31*), alongside the potential influence of social media, and the current limitations in the literature, it is crucial to deepen our understanding of how youths interact with and respond to social media feedback and its effects on their mood. To directly address this issue, we rely on computational analysis of Instagram trace data (i.e., real app posting and feedback data) and on an experimental study.

**Fig. 1.**
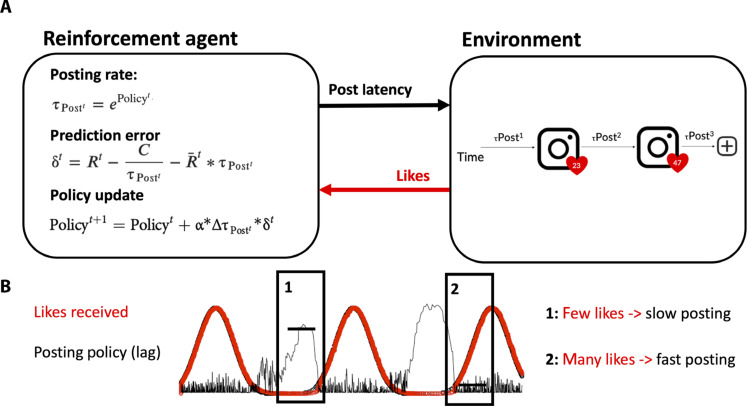
Computational approach. (**A** and **B**) The RL model states that the agent will select the latency until it will post again, τPost*^t^*, after it receives feedback on the current post. This posting latency is drawn from an exponential distribution determined by the current policy (Policy*^t^*). It posits that the agent’s posting latency is influenced by the number of likes received (e.g., 23 likes represented by the heart). The model states that the agent maximizes the reward rate by adjusting the policy (Policy update) after receiving a certain number of likes for a particular post. The model policy is adjusted on the basis of the learning rate (α), the change in posting latency (ΔτPost*^t^*), and the net reward prediction error (δ). The learning rate parameter indicates the sensitivity to social media feedback, and it is our prime variable of interest. The δ consists of the difference between the reward received and the average net reward rate (*R*) which considers both the effort cost associated with quick responses and the opportunity cost (or missed opportunities) of slow responding. The RL model was fit to individual Instagram trace data (see Fig. 2, A to C, for frequency distributions), where we estimated individual values for three free parameters: learning rate, α; effort cost sensitivity, *C*; and initial policy, ρ, based on maximum likelihood estimation. We focused on the two first parameters to test age differences in sensitivity to likes (α) and effort associated with posting (*C*; see Fig. 2, D and E).

**Fig. 2.**
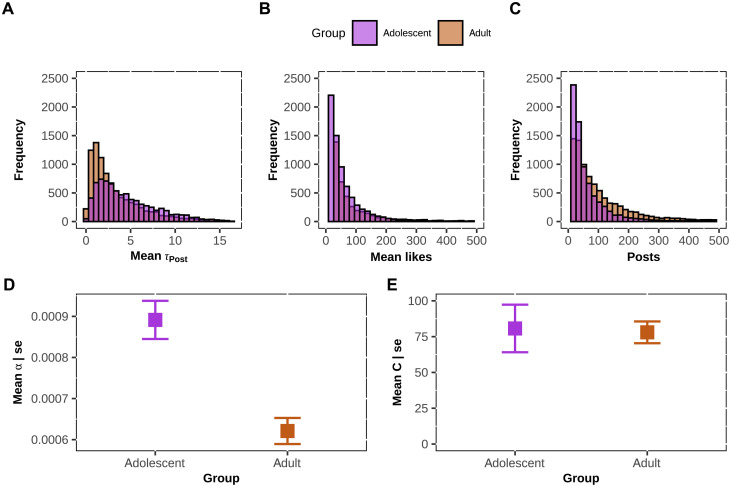
Social media engagement of adolescents is driven by sensitivity to social feedback. (**A** to **C**) Frequency distributions of Instagram trace data of adolescents (in purple) and adults (in orange). (A) Adolescents on average were slower with a posting latency of 2.20 days, while adults were faster and posted on average every 1.26 days. (B) Overall, adolescents had more likes for their posts with a mean of 207.5 likes compared to adults who had a mean of 97.28. (C) Adolescents posted on average 71 posts, while adults posted on average 132 posts. (**D** and **E**) Mean comparison of the model parameters between adolescents and adults: (D) Adolescents showed a significantly higher learning rate than adults, (E) but adolescents’ effort cost was not significantly different from that of adults.

**Fig. 3.**
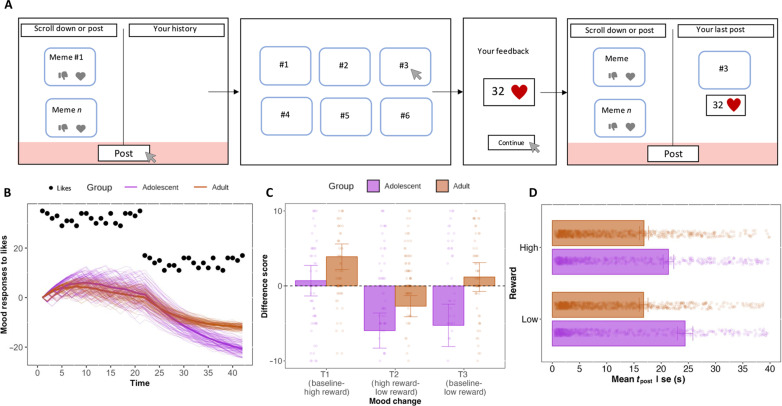
Adolescents feel more negative after a reduction in likes than adults. (**A**) Participants scrolled a meme feed and posted for 14 min. They posted by pressing “post” and selecting a meme from a set of 6. After posting, they received feedback (32 likes; fig. S3A) and returned to the main screen where they could scroll/post and see feedback for their last post. (**B**) Mood-RL simulations. Current mood (*m*^t^) is updated by *m^t+1^* = *m^t^* + η*^t^* (δ*^t^* − *m^t^*), where the prediction error (δ) is generated on the basis of the RL model. Mood (*m*) increases with positive δ (more likes than expected) and decreases with negative δ. The mood learning rate (η) was held constant for both groups, differing only in the core learning rate from the RL model (based on study 1 results). Adolescents show larger mood fluctuations (dark purple line; average mood change) than adults (dark orange line; average mood change) as likes change (dots). Faded lines show 100 simulations of mood change for both groups. (**C**) Mean mood to variations in likes. Jittered points represent single data points truncated for easy visualization (see fig. S3C for frequency distributions). (**D**) Mean response latencies (in seconds; ±1 SEM) to high and low rewards. Jittered points represent single data points truncated for visualization (see fig. S3B for frequency distribution).

We built our computational analysis on a validated computational reinforcement learning (RL) model (Fig. 1) (*15*), based on animal learning theory, which explains how foraging behavior is optimized through the balance between effort and opportunity costs, to ultimately maximize the average rate of rewards (*32*, *33*). The model posits a specific prediction of online engagement as a function of social feedback—in this case, likes. That is, the more likes a person receives, the sooner this person will post again, and vice-versa. In addition, the model assumes that there is an effort cost of posting (opening the app and creating content) that increases with the rate of posting (hence putting a limit on the posting rate).

As a result, this model provides two parameters that can explain individual or developmental differences in social media engagement: (i) the learning rate (α), capturing the sensitivity to social feedback; and (ii) the effort cost (*C*), which captures the effort associated with posting. By quantifying the sensitivity to social media feedback and the effort required to post, this framework provides a structured way to directly test age differences in the impact of social media feedback on social media engagement. On the basis of the reward sensitivity hypothesis (*34*), we expect adolescents to be more sensitive to social feedback and thus exhibit a higher learning rate compared to adults. In addition, given that adolescents are considered digital natives, we expect them to display a lower effort cost than adults.

## RESULTS

### Social feedback sensitivity on Instagram

In our preregistered study, we tested the hypothesis that adolescents would be more sensitive to social feedback and show a higher learning rate compared to adults in a large Instagram dataset (*35*) consisting of Instagram posts of adolescents (study 1: *n* = 7718, estimated ages: 13 to 19 years) and adults (*n* = 8895, estimated ages: 30 to 39; total_posts_ = 1.724.926; for details of the sample, see Materials and Methods). Instagram is a social media platform that is popular with both youths and adults and thus allows for the direct comparison in social feedback sensitivity. As expected, adolescents showed a significantly higher learning rate, that is, 44% greater [mean (*M*) α = 0.0009], compared to adults [*M*_α_ = 0.0006; Welch two-sample *t* test: *t*(13965) = 4.81, *P* < 0.001; with a small effect size *d* = 0.08; Fig. 2D]. However, adolescents did not significantly show an overall lower effort cost (*M*_C_ = 81) than adults [*M*_C_ = 78; Welch two-sample *t* test: *t*(10877) = 0.148, *P* = 0.88; Fig. 2E]. These differences survived numerous robustness checks and model specifications [see Supplementary Results (S1.1) and table S1 for details and model comparison]. Furthermore, given that posts can obtain multiple likes and make users less sensitive to social feedback, we also tested the utility function of likes [see Supplementary Results (S1.2) for more details]. Together, these data support the hypothesis that adolescents’ social media engagement is more strongly motivated by their response to likes compared to adults, and it is not related to their superior skills as digital natives (i.e., not the effort cost).

### Social media and mood: Experimental evidence

Adolescence is a period of heightened sensitivity to both rewards and social rejection, and the absence of expected social feedback could significantly affect individuals’ moods (for better or worse). Converging evidence from neuroscience studies suggests that mood may be represented as a running average of prediction errors (*36*, *37*); when individuals receive more likes than they expected, their mood will increase, and conversely, receiving fewer likes than expected will worsen it. This specific mechanism suggests that there is a direct link between the learning rate (sensitivity to likes) and mood changes. That is, higher sensitivity leads to greater mood variations in response to changes in likes. Hence, changes in social feedback could affect mental health by triggering constant shifts in mood (*38*). On the basis of simulations, we predicted that adolescents would show greater mood changes in response to a sudden change in likes received on social media (see Fig. 3B). More specifically, given that fewer or absence of likes may have a more profound effect on their mood, evoking negative feelings (*39*), we expected that adolescents would decrease posting behavior and report greater reduction in mood when confronted with a sudden decline in the number of likes compared to adults.

We used a preregistered online experiment mimicking features of social media platforms (Fig. 3A), such as Instagram, to investigate how social feedback would affect mood. In this experiment, adolescents (study 2: *n* = 92, ages: 16 to 20) and adult participants (*n* = 102, ages: 30 to 40) could scroll in a feed and post “memes” for which they receive real feedback (number of likes). To test the impact of changes in likes on mood, we manipulated the amount of likes participants could receive for their posts by providing them with different sets of pre-liked memes. In the high reward (HR) condition, participants received between 28 and 34 likes for their posts, and in the low reward (LR) condition, between 6 and 18 likes (see Materials and Methods for more details). Furthermore, participants reported their mood on three occasions: before the experiment started (T1), between HR and LR conditions (T2), and after the experiment (T3).

As expected, contrasting the HR and LR conditions, adolescents’ mood was more affected (T2: *M* = −5.96) by a decrease in the number of likes they obtained for their posts than adults (*M* = −2.71; *U* = 3875.5, *r* = 0.17, *P* = 0.036; Fig. 3C). Furthermore, while both age groups generally reported feeling more positive after the HR condition (T1; *M* = 0.70; *M* = 3.89, respectively; and did not differ at this initial point: *U* = 4115.5, *r* = 0.12, *P* = 0.14), adolescents concluded the experiment with a more negative mood (T3: *M* = −5.26) compared to adults who reported a positive mood (*M* = 1.19; *U* = 3867.5, *r* = 0.18, *P* = 0.03; Fig. 3C). However, posting latencies did not differ between reward conditions and age groups (interaction effect: *b* = 0.01, SE = 0.07, *z* = 0.16, *P* = 0.87), but adolescents posted less often (main effect of age group: *b* = 0.36, SE = 0.13, *z* = 2.75, *P* = 0.01; Fig. 3D). Together, these findings provide evidence that adolescents’ mood is more strongly affected by variations in social media feedback, particularly they experience more negative mood after receiving fewer likes compared to adults. This effect was independent of their self-reported problematic social media behavior or levels of social anxiety [see Supplementary Results (S1.3 to S1.4) and tables S3 and S4 for additional analyses and robustness checks; see also Supplementary Results (S1.5) for further analysis on sex differences].

### Social feedback sensitivity and individual differences in brain volume

**Fig. 4.**
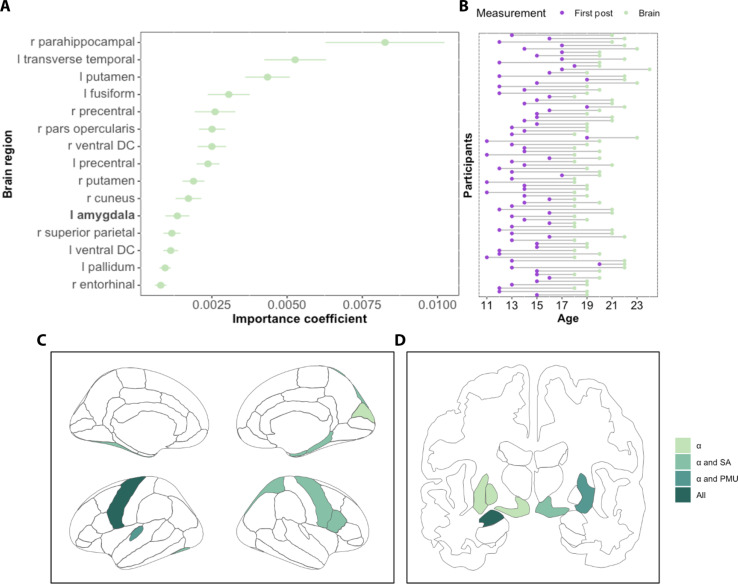
Brain regions associated with social feedback sensitivity, social anxiety, and problematic social media use. (**A**) List of both cortical and subcortical brain regions related to individuals’ sensitivity to social feedback on social media. The volumes of the 83 regions of the Desikan-Killiany [DK; (*73*)] atlas were extracted (*74*) from the T1 weighted scans using the FreeSurfer pipeline [see Supplementary Results (S1.9) for more details]. (**B**) Years of social media use per participant, spanning from the age of their first Instagram post (shown in purple) to the age at which brain data were collected (shown in green). (**C**) Cortical regions of the DK atlas (left hemisphere on the bottom and right hemisphere on the top) related to feedback processing. (**D**) Subcortical regions predicting social feedback sensitivity (α; light green) and overlapping regions between α and social anxiety (SA; right ventral DC), between α and problematic media use (PMU; right putamen), and across all (left amygdala). All these features performed better than the benchmark random feature.

Our next study added a set of exploratory neuroimaging analyses in a group of older youth (study 3; *N* = 96, emerging adults, ages: 18 to 24; Fig. 4, A to D). These participants provided their Instagram trace data (fig. S5, A to C, and table S7) consisting of historical social media data (with a mean of 5.74 years of use and mean age of first post at 14.2; Fig. 4B). These data allow us to focus on long-term effects of longitudinal social media use and identify which brain regions are associated with prolonged exposure to social media feedback. In addition, they filled in questionnaires on self-reported social anxiety and problematic social media behavior. Given our previous findings that adolescents were particularly sensitive to social media feedback, we were specifically interested in the brain regions that were associated with individual differences in learning rates. The sensitivity to social media feedback may be specifically related to the development of subcortical regions involved in feedback processing (*34*), regions that continue to develop during emerging adulthood (*40*). First, we fitted the computational RL model to the Instagram trace data to estimate learning rates [see fig. S6A for frequency distribution and Supplementary Results (S1.6) for model comparison]. Then, after extracting the volume of the 83 brain regions, we used random forest analyses with cross-validation (see Materials and Methods) to identify important brain regions associated with our main variable of interest (social feedback sensitivity: α). We used feature permutation importance, with a random variable as a benchmark, to determine the relevant brain regions. The advantage of the random forest method lies in its ability to identify regions that show both linear and nonlinear relationships, and the cross-validation enhances the robustness of our exploratory results.

As expected, we found that several subcortical regions involved in basic feedback processing are important in predicting social feedback sensitivity (α), such as the amygdala, ventral diencephalon (DC), pallidum, and the putamen, including additional cortical regions (see Fig. 4A). We further explored these findings by performing random forest analyses using social anxiety and problematic social media behavior as dependent variables to determine whether there were similar brain regions associated with social feedback sensitivity. Each construct showed its unique associations with social feedback sensitivity (Fig. 4, B and C), but the amygdala stands out as the single brain region that is associated with all three constructs (Fig. 4C; see fig. S7, A to C, for a full list of brain regions and tables S8 to S10).

## DISCUSSION

At a time of increased worries about the impact of social media on the youth, we focused on the most common affordance across all social media platforms, receiving likes. Across two studies using trace and experimental data, we found converging evidence suggesting that youths exhibit heightened sensitivity to social media feedback compared to adults. Replicating earlier work (*15*), we found that changes in likes may increase or decrease the motivation to engage with the platform. We extended these findings in two major ways: (i) we show that youths are more sensitive to likes and that (ii) changes in likes may result in greater changes in mood. Last, exploratory neuroimaging analyses suggest that the amygdala is a key region that is related to individual differences in sensitivity to likes, social anxiety, and problematic social media use. In the following sections, we unpack each of these results and discuss their broader implications.

Results from study 1 show that the social media engagement of adolescents is more strongly driven by their sensitivity to social media feedback and not just their dexterity in posting as digital natives. It is important to note that the higher learning rate indicates sensitivity not only to receiving more likes than expected but also to the unexpected absence of likes. Consequently, adolescents will engage more strongly with social media platforms if they receive many likes, but at the same time, they will also disengage more quickly when the likes are not forthcoming. Our findings are in line with theories that suggest that youths have a strong motivation to engage with social media to gain social validation (*13*, *41*, *42*) but are also affected more strongly by social rejection (*43*). Another potential mechanism influencing the observed behavior between both age groups is the perceived size and representativeness of the audience. Adults may be less responsive to likes compared to adolescents if their social media audience is smaller and less reflective of their broader social group outside social media. However, this does not exclude the interpretation that adolescent sensitivity to social feedback, well established in previous work (*44*–*48*), contributes to their stronger responses to social feedback compared to adults in similar experimental settings. This pattern is also observed in our study 2, where both adolescents and adults were given the same experimental manipulations involving social media feedback from unknown sources, and adolescents still exhibited stronger responses to variations in the number of likes than adults. Future research could benefit from examining the relative weights of different sources of likes. For instance, a like from a friend may carry more weight than a like from a random stranger. Nevertheless, enhancing awareness of available features, such as the ability to conceal likes on one’s account, an option implemented by Instagram in 2021, could markedly affect how youths engage with social media.

Next, we experimentally demonstrate that adolescents’ mood is more strongly affected by a reduction in the number of likes they receive on a social media simulator. This finding aligns with existing literature highlighting adolescents’ increased sensitivity to social approval and validation (*48*) and, therefore, may perceive the number of likes as a direct reflection of their social status and personal worth (*13*, *41*). Particularly, negative emotional responses to lack of positive feedback may be related to heightened rejection sensitivity (*49*), which, if experienced often, could potentially lead to mental health consequences (*50*, *51*). Adolescents’ sensitivity to social feedback is not unique to social media, as suggested by numerous studies in other contexts (*44*, *45*, *47*, *48*). This sensitivity and mood variability, in general, accompany their ongoing changes in multiple domains of their lives, such as biological, social, and psychological. Although our experimental study does not focus on or directly measure mood variability, our results indicate that specific patterns of social media feedback may contribute to increased mood variability in adolescence. During this period, individuals may thus be particularly sensitive to social media design features that provide immediate and quantifiable feedback. In contrast, adults may have a more established self-concept and be more resilient to such social media metrics.

Currently, adolescents spend a substantial amount of time on social media [e.g., with 45% of American adolescents saying they use the internet “almost constantly” (*52*)]. Given this extensive exposure to social media feedback, even small effects of likes on mood may significantly affect adolescents’ mood dynamics in daily life. The amount and frequency of social feedback are much higher than previously possible, creating a more intense environment compared to sporadic offline interactions. Given that even a small intervention can change mood and platform engagement, as our study suggests, it is crucial to further investigate the cumulative effects of feedback, especially since increased mood variability is a key predictor of future mood disorders (*51*). Our results underscore the urgency of acquiring more comprehensive data and methods, such as data donation and ecological momentary assessment, respectively, to fully understand the implications of social media feedback sensitivity on psychological well-being. Moreover, previous research has indicated sex differences in the relationship between social media use and well-being during adolescence (*53*, *54*). Future empirical work could investigate sex differences in mood responses to social media feedback as well as in the context of reward learning in social media behavior.

Results from our exploratory computational neuroimaging results revealed that individual differences in the volume of key subcortical regions of the valuation network (*55*) are associated with the sensitivity to likes. This supports the hypothesis that sensitivity to social media feedback in youth is, to some extent, related to developmental differences in brain structure and function (*34*). Furthermore, studies on broader social interactions have shown that social feedback in the form of exclusion and rejection is related to neural responses in the anterior cingulate cortex, right ventral prefrontal cortex, and amygdala (*56*, *57*), which may be similar in face-to-face interactions and virtual environments (*34*, *58*). Social feedback in the form of acceptance is related to youths’ heightened activation in the ventral striatum and prefrontal cortex (*59*, *60*). In addition, habitual social media checking behaviors among adolescents may be associated with changes in neural sensitivity to the anticipation of general social rewards and punishments which could have implications for psychological adjustment (*6*). Our findings support the notion that there may be some overlapping neural mechanisms, including key regions of the limbic and valuation network regions, underlying offline and online social feedback sensitivity.

Last, our analyses revealed that the amygdala is a key region involved in processing social media feedback, and it is related to individual differences in problematic social media use and social anxiety. While our results suggest that the amygdala is involved in these processes, it is important to note that this does not imply direct causation, and these processes were also associated with distinct networks of regions. The amygdala’s role in processing emotional responses can contribute to different outcomes depending on individual circumstances and contexts. In addition, we emphasize that we focused on individual differences in normative social anxiety rather than on clinical cases. Future research should include fine-grained developmental windows and longitudinal data to investigate how youths’ sensitivity to online social feedback is associated with the development of subcortical valuation regions.

In our analyses, we put our focus on one of the most salient, and seemingly innocent, affordances that is common across almost all social media platforms, receiving likes. This focus already allowed us to show meaningful developmental variance in an important cognitive mechanism, but of course, social media platforms provide more complex and qualitative opportunities for feedback. A logical next step would be to focus on comments, which are among the most common affordances of social media, offering a potentially more nuanced way of providing feedback. Previous research has already shown that the level of affect associated with messages and comments increases their impact (*61*). Combining automated sentiment analysis (*62*) could enable the extension of computational analysis to integrate the impact of comments, including their valence and effect. The current computational framework is an example of and could be integrated into the broader effort to use multimodal data sources. This would help develop precise computational phenotypes to identify the multitude of different social media types (*28*) and gain deeper insights into the types of users and use to be able to provide tailored interventions (*63*).

In sum, our results suggest that the current design of social media platforms, characterized by immediate quantified social feedback, may be more impactful for youths. This is a crucial period marked by heightened sensitivity to peer approval and rejection, and the current prevalence of likes as a proxy for online approval fosters a culture of comparison and validation-seeking behavior. It is also a period of rapid socio-emotional changes that can affect long-term mental health and well-being (*64*). Our findings highlight the importance of considering age-specific user policies and strategies in the design of social media platforms, suggesting two avenues for intervention. First and foremost, platforms should change incentive structures, such as shifting the emphasis away from likes to more meaningful engagement; the possibility of hiding likes is an interesting step in this direction. Second, our results suggest that we should not only focus on strengthening the digital literacy of youth, a generation who may often be more literate anyway but focus on developing skillful emotion regulation in online environments. Here, the constructs of digital competence (*65*) and digital maturity (*66*) are helpful tools. Social media–generated emotions can occur frequently, at any time, and even unnoticeable to others. An approach addressing emotion regulation skills online may be crucial for youth to deal with the constant variation of feedback they are exposed to on social media.

## MATERIALS AND METHODS

### Social media data

The data of study 1 was based on two datasets published by a previous study (*35*) in which the data were originally collected for 6 months between October 2014 and March 2015, using the Instagram Application Programming Interface (API). These two datasets consist of Instagram data from two developmental groups: adolescence (estimated ages: 13 to 19) and adulthood (estimated ages: 30 to 39). Initially, data from 2 million random Instagram users were collected. These data were further reduced to 10,000 active users per age group based on several automated heuristics and manual verification by human judges [see (*35*) for a thorough procedure]. The datasets were made available after an agreement had been signed for the use of the Instagram data. Given the purposes of our study, we focused on the datasets that originated from the verified profile-based and tag-based samples including 10,000 adolescents and 10,000 adults. These datasets contained data pertaining to users’ activities, such as number of posts, timestamps (date of the post), and number of likes. On the basis of the procedure used by (*15*), we excluded individuals with less than 10 posts and those cases in which the timestamp was not available. The final datasets consisted of 1.724.926 posts from 7718 youth and 8895 adult Instagram users.

Study 2 was conducted online, and we recruited participants from English-speaking countries via Instagram to participate in a study about social media. A total of 211 participants (102 adolescents and 109 adults) completed the study, but after data exclusions, the final sample consisted of 92 adolescents and 102 adults (see data exclusion criteria). Participants were compensated with a $5 voucher for their participation in the study, and a participant from each age group was randomly selected to win a $50 voucher.

Study 3 was part of a larger project on social learning and social media use and consisted of a survey completed online and a magnetic resonance imaging (MRI) scan at the Spinoza Centre for Neuroimaging. A total of 106 participants provided their Instagram trace data (posts, timestamps, and number of likes) but also self-reported social anxiety, problematic social media use, and structural brain data. We applied the same criterion as in study 1 and kept only those individuals who had more than 10 posts. This led to a sample reduction of 106 users from whom we had access to Instagram trace data to 96 users and 11,277 Instagram posts. Furthermore, in 2015, Instagram introduced the carousel post which allows the user to post 10 single pieces of content (e.g., images) simultaneously. Although this type of post counts each piece of content as an individual post, the carousel post contains the same number of likes for each post as well as the same timestamp (date of the post; being the posting latency 0 between all individual posts of the carousel post). To make it comparable to trace data in study 1, for each user having this type of posting, we kept only the first post and retained the corresponding number of likes and timestamps. Moreover, these data consist of user historical Instagram trace data integrating all the data since participants created their accounts. This resulted in an average of 5.74 years (SD = 1.96) of social media use among participants and an average age of first post at 14.2 (SD = 2.08). We therefore modeled data that overlaps considerably with the ages of studies 1 and 2. Although structural brain data were collected when individuals were slightly older (age range: 18 to 24) than participating groups in studies 1 and 2, the observed brain structure differences are related to sensitivity to social media feedback over the period of adolescence. Participants provided their informed consent for participation and received a monetary compensation of €25 to complete the study. The current work was approved by the Ethics Review Board Faculty of Social and Behavioral Sciences of the University of Amsterdam (study 1: 2021-DP-13838; study 2: FMG-2485_2023; study 3: 2019-DP-10814) and was performed in accordance with relevant guidelines and regulations.

### Study 1

#### Model recovery and power analysis

To develop our analysis plan and test our hypothesis that youths are more sensitive to online social feedback than adults, we first simulated two datasets generated by the same process. This simulated data only differed in the average learning rate (α) to generate predictions for reward sensitivity between the two developmental populations. To simulate the behavior of our adult group, we drew values for the learning rate from a truncated normal distribution with a mean of 0.002 and SD of 0.002 (truncated by excluding values < 0). In addition, for each simulated agent, we drew a value for the cost function from a uniform distribution between 0 and 1, and the number of observations was drawn from a normal distribution with a mean of 70 and SD of 20 (close to the individual mean posts of the adult empirical dataset). On the basis of these settings, we generated 1000 simulated adult agents. To simulate the adolescent dataset, we changed the mean of the generative distribution for the learning rate to 0.003, keeping all other parameters constant. Our first step was to use these simulations to determine model recovery and how such a minimal increase in learning would lead to a detectable difference in the learning rate between the two developmental groups (by performing power analysis; see fig. S1). After fitting the model, we found that both the learning rate and cost parameters were recovered for the full dataset [*r*(1959) = 0.18, *P* < 0.001 and *r*(1959) = 0.36, *P* < 0.001, respectively; fig. S2]. Yet, the most important question was whether the model could reliably recover the group differences in the learning rate and, if so, use that to establish the minimal sample size for our empirical data. As expected, we found differences in the learning rate [adult group: mean α = 0.00238; adolescent group: mean α = 0.00294; *t*(1959) = −7.47, *P* < 0.001], but no differences were found in the estimated value for the cost parameter [*t*(1957) = −0.98, *P* = 0.33].

### Study 2

#### Experiment

The experiment was conducted online, and the recruitment of participants was done via Instagram. Participants provided their informed consent for participation before the start of the study. Overall, the study took between 20 and 25 min (14 min for the experiment and the remaining minutes for instructions and for postexperiment questions about demographics, social media behavior, and social anxiety). Participants were informed that the memes they could select from to post were previously rated by 40 people with a like, neutral, or a dislike on a survey in Qualtrics. For the purposes of the current study, we focused on likes.

The experiment resembled key aspects of a social media platform, such as Instagram. Participants, on one hand, were able to see and like or dislike humorous images (i.e., memes) in a continuous feed such that they could scroll down and see content as they wished. The feed contained 410 memes with no feedback (number of likes) visible to participants. These memes were different from those memes in the reward conditions, but they have been also rated previously with a distribution of between 18 and 28 likes. This allowed us to make a stronger difference in the set of memes between HR and LR conditions. To post, participants could press the button “post” which was always visible on the main screen (see Screenshots of the experiment). When participants decided to post, they could select a meme from a set of six different memes. This was done to prevent participants from creating inappropriate or unethical content while still giving a sense of self-expression. Participants were informed during the instructions that the memes they could select from in each step had been previously rated by 40 people in a prior experiment. Hence, they were aware the feedback was real. The experiment consisted of two blocks following the same order across all participants: first, the HR condition in which the pool of memes had between 28 and 34 likes and, second, the LR condition with memes having between 6 and 18 likes. Participants could see the real feedback (number of likes) for a particular meme every time they posted. Each condition took 7 min to complete. Before the experiment started, participants had the opportunity to try the feed for a minute. In addition, we asked participants to try to post four times so they would get familiarized with posting, and everyone would start with the same posting baseline. Posting response latencies were measured by tracking how much time (in milliseconds and converted to seconds) participants spent interacting with the feed before posting.

Furthermore, participants reported their mood and how they were feeling at that moment (1 = extremely negative to 100 = extremely positive) on three occasions: baseline (before the experiment), after the HR condition, and after the LR condition (end of the experiment). Last, we included postexperimental questions to measure participants’ self-reported social media behavior and well-being to test for group differences in mood change as an exploratory analysis. We focused on participants’ social anxiety (fig. S4, A and C) and problematic social media use (fig. S4, B and D).

#### Participants and power analysis for reward conditions

We simulated a dataset to determine the required sample size to run our experimental design and detect whether the impact of reward conditions (high versus low) on posting behavior depended on the age group, using a multilevel linear mixed effect model from the *simr* package (*67*). We defined a sample of 100 participants per age group (adolescents vs adults) and each participant had both HR and LR conditions. We defined the following parameter values for fixed effects based on reasonable effect sizes: intercept = 0.5, reward = 0.4, age_group = 0.2, and reward_condition*age_group = 0.3, as well as a random intercept for the subject of 0.1. On the basis of a power analysis, a total sample of around 200 participants (100 per age group) would ensure enough power.

#### Data exclusion and quantities of interest

We excluded data from participants who reported to be aged below 18 years old from countries in which informed consent cannot be provided by oneself if they are 18 years old or younger. Furthermore, we excluded from the main analysis participants who exited the experiment window for a considerable amount of time (we used 10% of the time of the experiment: 1.4 min as a criterion). This resulted in a final sample of 92 adolescents and 102 adult participants.

We were particularly interested in the participants’ mood and their posting behavior. We quantified mood as the difference score between measurement occasions in each age group (T1 = baseline–HR condition; T2 = HR condition–LR condition, and T3 = baseline–LR condition). Because of the limited number of trials within the subject, our experimental data would not allow for the implementation of a reinforcement learning model including mood changes (if we apply the rule of at least 10 posts per individual to allow for learning analysis, the sample would be reduced to 60 adolescents and 80 adults). Hence, we aimed for model-free analyses. We quantified response latencies from the first time a participant posted. Response latencies were computed as the time between two consecutive posts on the task. As comparable to real-world Instagram data (study 1), we also characterized the first latency as undefined and performed a multilevel generalized linear mixed model specifying a Gamma distribution with a log link function, using the *glmmTMB* package (*68*).

### Study 3

#### MRI data acquisition

Structural imaging data were acquired on a 3.0 T MRI scanner (Philips Achieva DS, 32-channel head coil) at the Spinoza Centre for Neuroimaging. The scan consisted of two high-resolution T1-weighted anatomical scans (0.70 mm by 0.81 mm by 0.70 mm, FOV = 256 mm by 256 mm by 180 mm, matrix size = 368 by 318 by 257 slices, TR = 11 ms, TE = 5.2 ms, parallel acquisition technique = SENSE), which were averaged.

#### MRI data preprocessing

The preprocessing and quality control of the MRI scans were done by automatic preprocessing using the *fMRIPrep* pipeline (fMRIPrep version 1.5.4). This procedure included artifact removal, cortical surface generation, skull-striping, cross-modal registration, and standard space alignment. The obtained T1-weighted images were corrected for intensity nonuniformity with N4BiasFieldCorrection (*69*), distributed with ANTs 2.2.0 (*70*) (RRID:SCR_004757), and used as T1w reference throughout the workflow. Next, this reference was skull striped with a Nipype implementation of the antsBrainExtraction.sh (from ANTs), using OASIS30ANTs as the target template. Brain tissue segmentation of cerebrospinal fluid (CSF), white matter (WM), and gray matter (GM) was performed on the brain-extracted T1w using fast [FSL 5.0.9, RRID:SCR_002823; (*71*)]. Volume-based spatial normalization was implemented, and the T1-weighted images were registered to one standard space (MNI152NLin2009cAsym) through nonlinear registration with antsRegistration (ANTs 2.2.0), using brain-extracted versions of both T1w reference and the T1w template. For spatial normalization, the template selected was ICBM 152 Nonlinear Asymmetrical template version 2009c (*72*) (RRID:SCR_008796; TemplateFlow ID: MNI152NLin2009cAsym; see workflows in *fMRIPrep*’s documentation for further details of the pipeline).

#### Gray matter volume extraction

GM volumes of cortical and subcortical brain areas of both hemispheres corresponding to the Desikan-Killiany atlas (*73*) were extracted (*74*) using FreeSurfer (http://surfer.nmr.mgh.harvard.edu/). GM volumes of 89 areas were scaled to account for brain size using the *SupraTentorialVolNotVent* parameter, which includes GM and WM volumes of the brain (excluding cerebellum, brain stem, ventricles, CSF, and choroid plexus). Given that the size of the ventricles impacts WM and GM volumes, the ventricles were subtracted from the total brain volume. In addition, to prevent collinearity between brain regions, the volume of brain areas that occur in both hemispheres was averaged if the hemispheres were highly correlated (i.e., Pearson’s correlation of 0.7 or higher). This led to the reduction of 6 brain regions and a final number of 83 brain regions included.

#### Feature extraction

We ran random forest regression and used feature permutation importance to identify relevant brain regions related to social feedback sensitivity, social anxiety, and problematic media use. The random forest was trained using *sklearn* version 0.24.2 in Python (*75*). The random forest regression fits several decision tree classifiers on several subsamples of the dataset by applying bootstrapping and uses a different set of features for each decision tree. Each of these individual random forest trees produces a prediction which is averaged into a final prediction. Before training each model to predict the learning rates, social anxiety, and problematic media use separately, we normalized all features by scaling them between 0 and 1 and making them comparable. Furthermore, we applied a leave-one-out cross-validation (LOOCV) outer loop, where one participant in each loop was used to evaluate the model, which was trained on the remaining participants (*n* − 1). This led to one model per participant in the case of learning rate (*n* = 76), social anxiety, and problematic media use (*n* = 84). To increase reliability and robustness, we set the number of decision trees to 1000 but kept the other hyperparameters at default. We used permutation importance (*76*) because it is a reliable method. It works by randomly shuffling each predictor variable to check how it affects the model accuracy. We averaged permutation importance across five permutations to account for random values being meaningful by chance. Last, this procedure computed a baseline feature “random” consisting of values between 0 and 1 not relevant to predict learning rates, social anxiety, and problematic media use. The random feature is newly computed for each loop within the LOOCV; and those features (i.e., brain regions) that are more important than the random feature (higher importance coefficients compared to random feature) were considered.

#### Self-reported measures: Study 2 and study 3

##### Problematic social media use

Participants completed an adapted version of the Compulsive Internet Use Scale (*77*). This version kept the items replacing “internet” with “social media.” An example question is “Do you find it difficult to stop using social media when you are online?”, and items are assessed on a five-point scale ranging from 1 = “never” to 5 = “very often.”

##### Social anxiety

Participants completed the Interaction Anxiousness Scale [IAS-3; (*78*)]. An example question is “I often feel nervous even in casual get-togethers,” and items are assessed on a five-point scale ranging from 1 = “not at all” to 5 = “extremely.”

### Statistical analysis

Main analyses were conducted in R statistical software, Rstudio v.1.3.1093 (*79*). We performed a power analysis of our main analysis in study 1 (independent sample *t* test, one-sided; for preregistered hypotheses and analyses: https://osf.io/mt2nv/?view_only=6e232108b6754961b783a9e98c042f3a). We computed the effect size (Cohen’s *d*) by calculating the difference between the means of the generated learning rates across both datasets and dividing it by the pooled SD. The main analysis in study 1 was conducted using the Welch two-sample *t* test which is robust in the case of large sample sizes. As a robustness check, we additionally computed a nonparametric test (see S2.1 for more details). In study 2 (preregistration: https://osf.io/q2htd/?view_only=ce5582b6d9414f1db552bb83c8d69b66), we simulated a dataset to determine the required sample size to detect the main effect of reward condition by age group in a multilevel linear mixed effect model. We computed the mood difference score between all measurement occasions in both age groups. We were particularly interested in the mood responses between HR and LR conditions (T2). To test mean differences in mood responses between age groups, we performed a Mann-Whitney *U* test for each time point given the sample size and non-normal distribution nature of the data. To test whether the effect of reward condition on post-latencies was dependent on the age group, we conducted a multilevel generalized linear mixed model specifying a Gamma distribution with a log link function. In study 3, we observed a few outliers, so we winsorized the learning rates before running the random forest to test for the associations with brain regions.

## Supplementary Materials

### This PDF file includes:

Supplementary Results Figs. S1 to S7 Tables S1 to S10 Screenshots of the experiment References
