# Supplementary material for: Youths’ sensitivity to social media feedback: A computational account
Source: Sci Adv. 2024 Oct 23;10(43):eadp8775. doi: 10.1126/sciadv.adp8775 (PMC11498218; doi:10.1126/sciadv.adp8775)
Supplement: Supplementary file 1 — Supplementary Results Figs. S1 to S7 Tables S1 to S10 Screenshots of the experiment References [file sciadv.adp8775_sm.pdf]

Science Advances
AAAS

Supplementary Materials for

Youths' sensitivity to social media feedback: A computational account

Ana da Silva Pinho *et al.*

Corresponding author: Ana da Silva Pinho, a.f.dasilvapinho@uva.nl

Sci. Adv. **10**, eadp8775 (2024)
DOI: 10.1126/sciadv.adp8775

This PDF file includes:

Supplementary Results
Figs. S1 to S7
Tables S1 to S10
Screenshots of the experiment
References

S1 Supplementary Results

This section consists of robustness checks, model comparison and results from additional analysis.

S1.1 Robustness checks and model comparison – Study 1

Outliers can bias hypothesis tests, particularly if individuals in our sample have extreme average response latencies (*τPost*) and low or high number of posts. As a robustness check, we removed all individuals that fall outside of the 20th – 80th deciles on either variable, and compared the means of the developmental groups in the remaining datasets. We obtained similar results after removing individuals from both groups who had extreme average response latencies and low or high number of posts (adolescents showed a higher learning rate (M = 0.0008) than adults (M = 0.0005; Welch two-sample t-test: $t(7767.9) = 6.2336$, $p< 0.001$)), confirming that the difference in sensitivity to online social feedback is not dependent on extreme cases.

Although the parametric test is quite robust to large sample sizes when data is not normally distributed, as another robustness check we ran a non-parametric test to compare both groups' means and obtained similar results (U = 38515525, r = -0.12, $p< 0.001$).

Model comparison was done using the Akaike information Criterion (AIC; (*80*)) which balances goodness-of-fit and model complexity, neutralising complexity differences between models and it is quite robust with large sample sizes. Model comparison indicates that the RL model performed better at describing social media behaviour on Instagram across adolescent, adult and collapsed data than a null model without reward learning (see Table S1 for AIC results). The null model assumes a fixed posting strategy (i.e., average response latency) on social media which is not influenced by online social feedback.

S1.2 Exploratory: comparison of utility function of likes in RL – Study 1

In the RL model, the utility of likes followed a linear identity function: $u(R)$ = R. However, posts on Instagram can receive several likes which may lead the user to some sort of habituation to online social feedback. In order words, the value given to a like may be different depending on the number of likes received for a post. Therefore, we further investigate the utility of likes on Instagram following an exponential function: u(R) = R^{d} by including a free parameter d ($0 \leq \boldsymbol{d} \leq \infty$) to the RL model (*16*). For instance, if a user with d = 0.5 receives 10 likes, the utility of that like is around ~3, while for 10000 likes the utility is ~100. Adding this free parameter improves

the RL model describing social media behaviour (Table S1 for AIC results) across adolescent and adult data. This finding is in line with (*15*), suggesting that individuals with a larger number of followers on Instagram may habituate to likes as they tend to assign less weight to each like and thus in need of more likes for an equivalent rewarding effect. We further tested this using linear regression to predict the number of followers (log-transformed) from the model estimated *d* parameter, controlling for the number of posts in the adolescent and adult data. We observed that a lower model estimated *d* parameter (more strongly diminishing marginal; utility of likes) was related to greater follower count in adolescents (b = -0.15, SE= 0.011, t = -13.12, p< 0.001) and adults (b= -0.20, SE= 0.012, t = -17.12, p< 0.001).

Additionally, we tested the differences in sensitivity to likes between adolescents and adults using the RL in which likes follow an exponential function. We obtained similar results to those reported in the main text: adolescents (M_{α} = 0.0014) are more sensitive to likes than adults (M_{α} = 0.0006; Welch two-sample t-test: $t(9039) = 6.59$, p< 0.001; with a small effect size d = 0.11). We also ran a non-parametric test to compare both groups' means and obtained similar results (U = 38921573, r = -0.13, p< 0.001).

S1.3 Robustness checks – Study 2

We removed individuals (n = 4) reporting mood values lower and higher than 1.5 times the interquartile range and reran the analysis. The results obtained after removing outliers were similar to results reported in the main text for each time point. No mood differences between age groups were found between at T1 (U = 3903.5, r = 0.13, p =0.1181), however adolescents were more negative at T2 (U = 3553, r = 0.21, p = 0.01275) and T3 (U= 3516, r = 0.22, p = 0.009664) compared to adults. Therefore, the differences in mood changes between adolescents and adults were not driven by these outliers.

S1.4 Social anxiety and problematic social media use – Study 2

Overall, adolescents reported to feel moderately socially anxious (M = 3.1; Fig. S4a) compared to adults who reported lower levels of social anxiety (M = 2.6; Fig. S4c; U = 5764.5, r = -0.23, p = 0.01). Moreover, both groups showed moderate levels of problematic social media use (adolescents: M = 3.2; Fig. S4b adults: M = 3.0; Fig. S4d; U = 4988, r = -0.06, p = 0.449). Although we did not find significant differences between adolescents and adults in their levels of problematic

social media use, adults reported slightly higher rates of problematic use. A possible explanation for this is that self-reported measures, in particular problematic media use, require metacognition to recognize a potential problem, which may be less evident in adolescent groups as they are developing their metacognitive skills (*81*, *82*). Additionally, adolescents might view high levels of social media use as normative because their peers may also use it extensively, whereas adults may have a lower threshold and thus report more problematic use.

Previous research suggested that social anxiety is related to mood (*83*) while problematic social media use is related to mood and addictive-like behaviours (*84*). During adolescence, individuals experience significant changes in mood (*85*), so we aimed to understand the relationship between social anxiety and mood changes as well as problematic social media use and mood changes during the experiment. However, we did not find evidence supporting a link between social anxiety and mood changes nor self-reported problematic media use and mood change across age groups (see Table S3 and S4 for regression results, respectively). This discrepancy could be due to differences between subject and objective measures of media use as highlighted by previous research. For instance, subjective measures of social media use showed a weak association with more objective usage data collected from devices (*27*, *86*). The scale of problematic social media use included in our study measures the perceived lack of control over time spent on or thinking about social media. Given the developmental differences in metacognition mentioned earlier, participants could have over or underestimated their usage, potentially limiting our results.

S1.5 Sex differences in mood change – Study 2

Although not the focus of our main analyses, in light of previous research suggesting sex differences in the relationship between social media use and well-being during adolescence (e.g.,(*53*, *54*), we conducted further analyses to investigate potential mood changes across sexes. For instance, female adolescents seem to experience an increase in mental health difficulties (e.g., anxiety, depressive symptoms) related to the onset of puberty (*87*) and they may spend more time on social media than their male counterparts (*88*). We ran a linear mixed-effects model separately for adolescents and adults to examine the sex effects across all mood measurements during the experiment. We did not find evidence supporting differences in mood change between females and males in the adolescent and adult groups (see Table S5 for descriptive statistics and Table S6

for regression results). However, given that we did not predict sex differences, our study was not designed to test such an effect. Therefore, these findings should be interpreted with caution due to the small and unbalanced sample sizes, which may affect the results limiting their robustness and generalizability.

S1.6 Model comparison – Study 3

As observed in Study 1, model comparison suggested that the RL model (AIC = 67390) accounted better for the time post decision than the null model without reward learning (AIC = 69880).

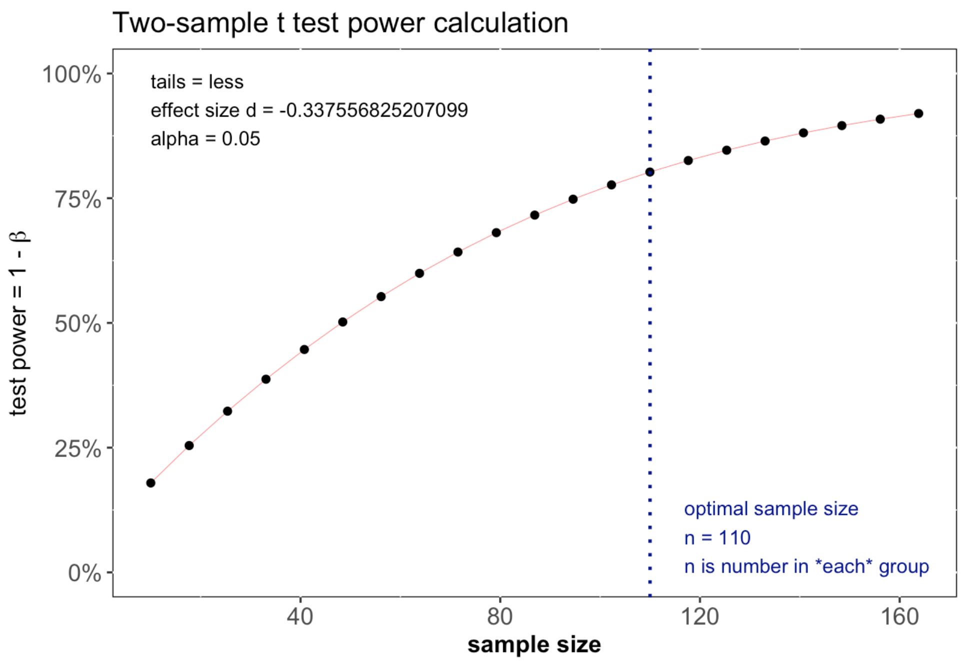

Fig. S1. Power analysis.

We performed power analysis using the function *pwr.t.test* of the *pwr* package (*89*). The performed power analysis for our main analysis of Study 1 indicates that we would need a minimal sample of 110 individuals in each group to find a reliable difference in learning rates (at power level of 80% and at $\alpha = 0.05$). Given that we include in our sample over 7000 adolescents and over 8000 adults and we focused on a very small deviation of the learning rate, we expect that our sample is of an adequate size.

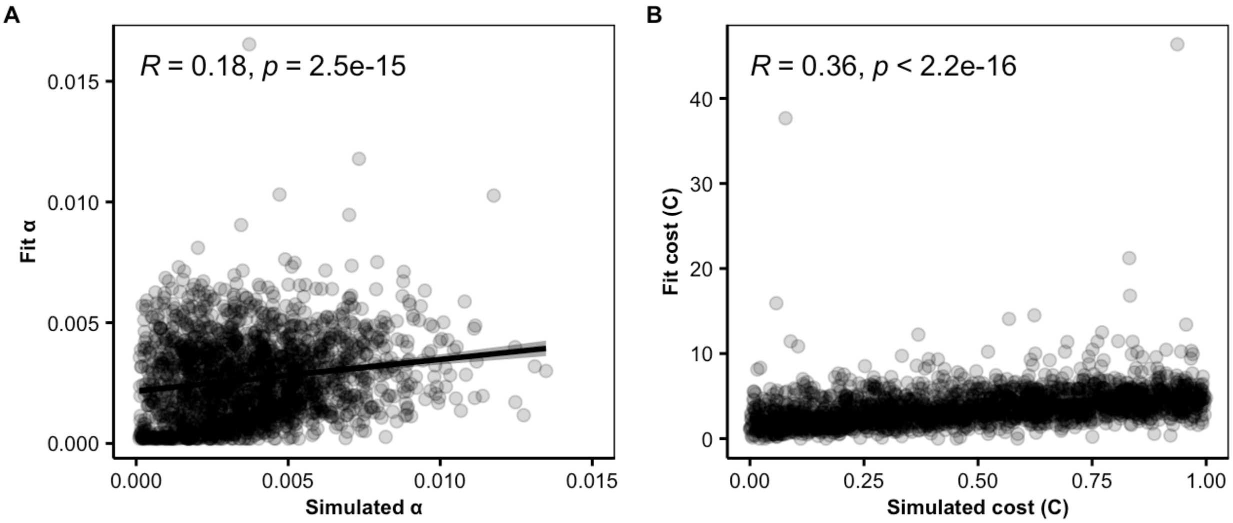

Fig. S2. Model recovery of parameters of interest.

(**A**) Learning rates and (**B**) effort cost of the generated datasets were recovered.

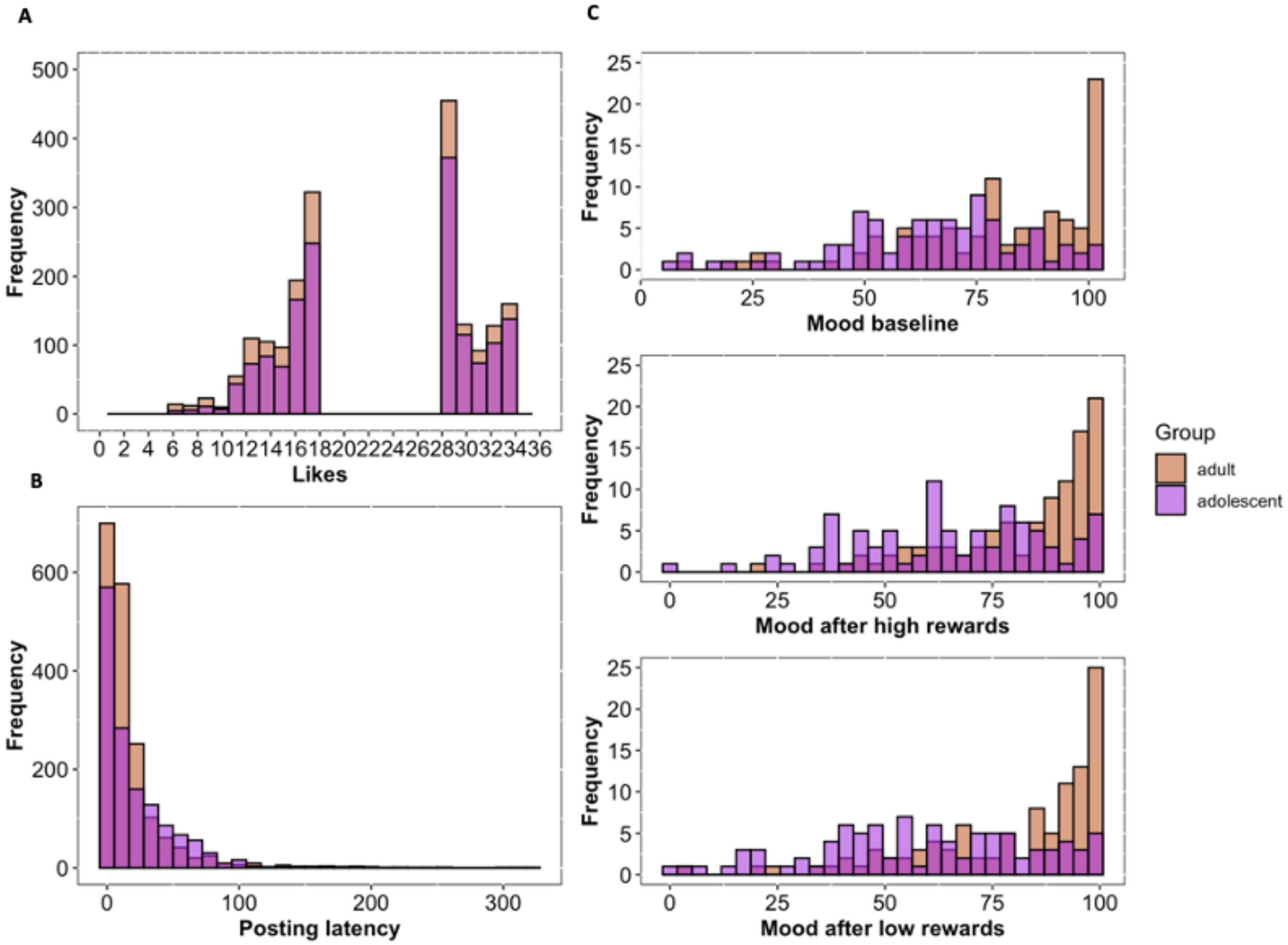

Fig. S3. Frequency distributions of behavioural measures.

(**A**) Frequency distribution of the number of likes adolescents (purple) and adults (orange) received for their posts (adolescents $total_{posts} = 1524$; adults $total_{posts} = 1924$) in the low reward condition (on the left) and high reward condition (on the right). (**B**) Frequency distribution of response latencies. Overall, adolescents' responses were slower than adults (adolescents $M_{High} = 21.3$s and adults $M_{High} = 16.8$s; Welch two-sample *t*-test: $t(1492.9) = 3.50$, $p < 0.001$; adolescents $M_{Low} = 24.4$s and $M_{Low} = 16.8$s; Welch two-sample *t*-test: $t(1154.1) = 4.66$, $p < 0.001$). (**C**) Adolescents reported to be more negative than adults particularly after the low reward condition.

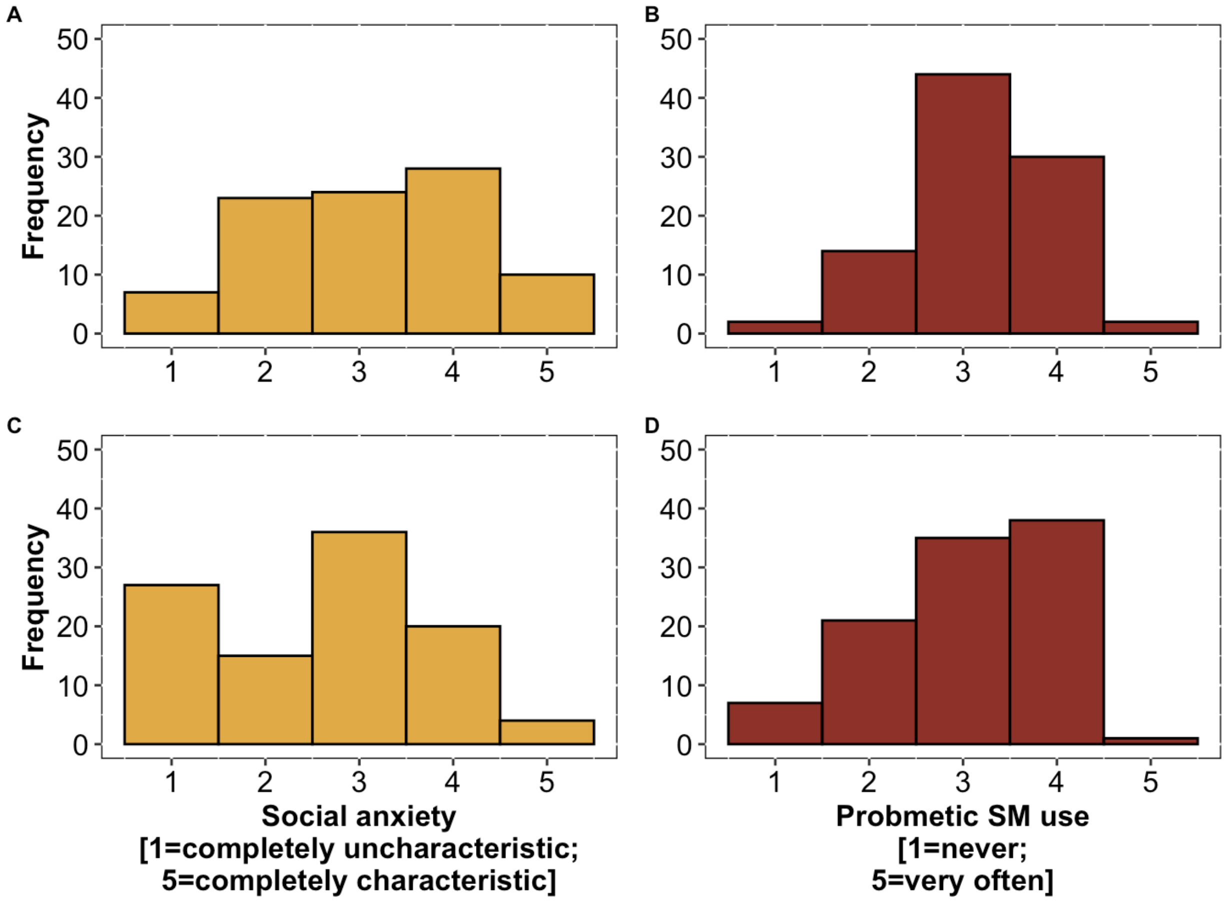

Fig. S4. Frequency distributions of self-reported measures.

(**A-D**) Self-reports of adolescents on (**A**) social anxiety (on a 5-point scale ranging from 1 = 'not at all' to 5 = 'extremely') and (**B**) problematic social media (SM) use (on a 5-point scale ranging from 1 = 'never' to 5 = 'very often') (**C-D**), self-reports of adults on the same measures mentioned in **A-B**.

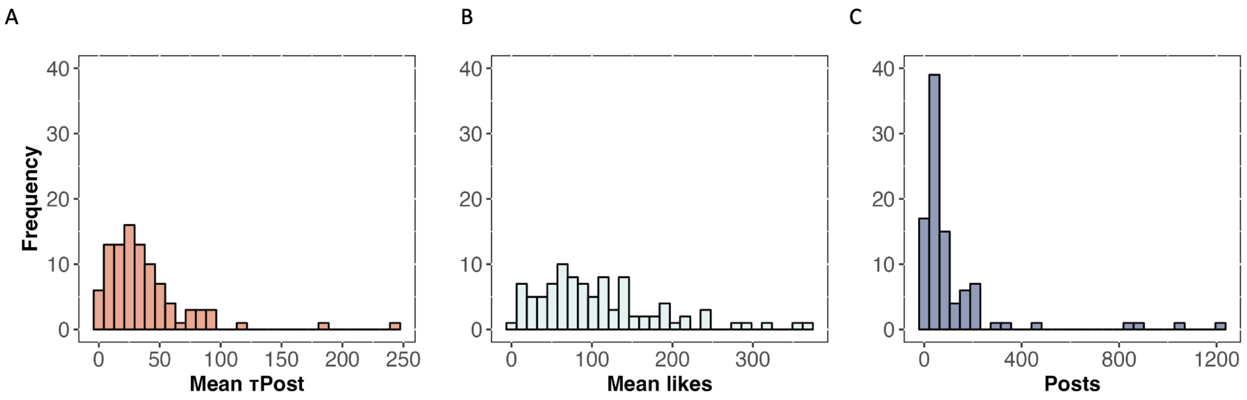

Fig. S5. Frequency distributions of self-reported measures.

(**A-B**) Frequency distributions of Instagram trace data of emerging adults. (**A**) Generally, their posting latency was of 14.5 days, (**B**) with an average of 89 likes (**C**) and average of 117 posts.

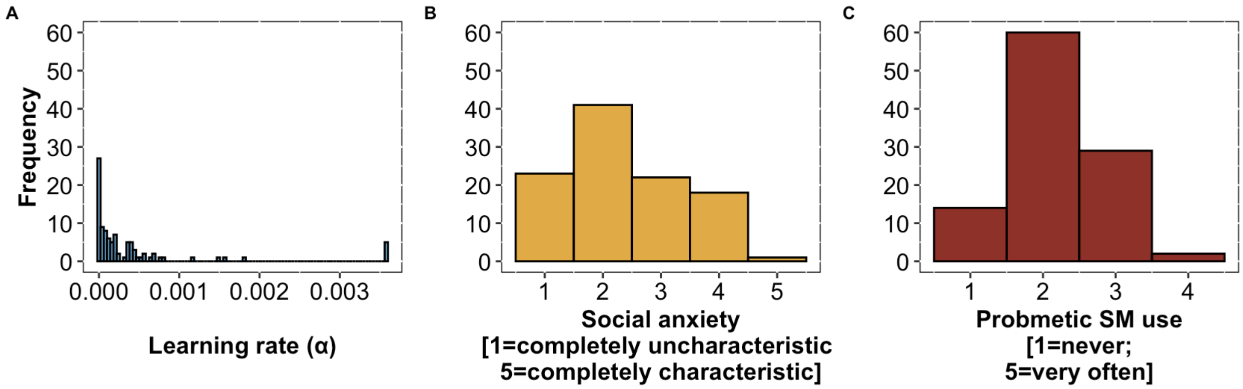

Fig. S6. Frequency distributions of sensitivity to social feedback, social anxiety, and problematic social media use.

(**A**) Frequency distribution of learning rates among a sample of young adults (N = 96) with population mean of $\alpha = 0.0004$. (**B**) frequency distribution of individuals' self-reported levels of social anxiety. Overall, individuals (N = 105) indicated relatively low levels of social anxiety ($mean_{social\ anxiety} = 2.4$). (**C**) frequency distribution of individuals' self-reported problematic social media (SM) use. Overall, individuals (N = 105) indicated relatively low levels of problematic media use ($mean_{media\ use} = 2.2$).

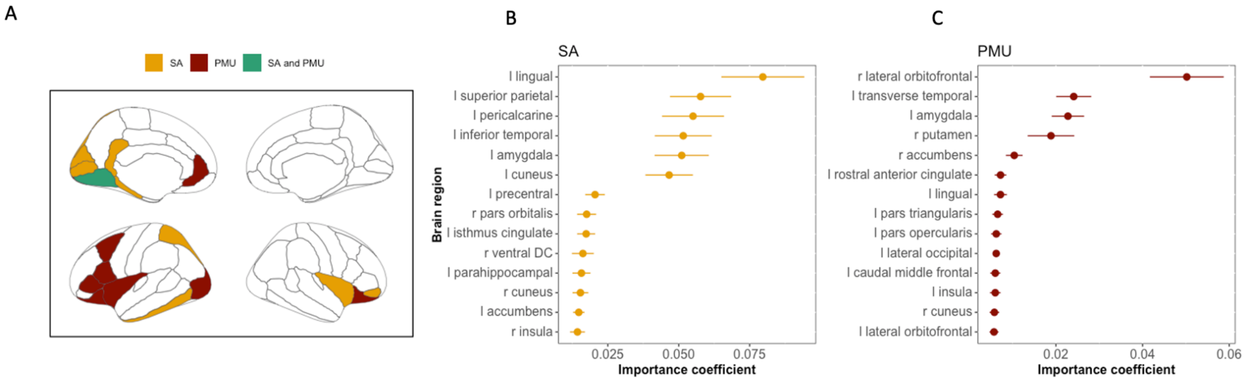

Fig. S7. Full list of most relevant features.

(**A**) Cortical regions of the DK atlas (left hemisphere on the bottom and right hemisphere on the top) related to social anxiety (SA; yellow), problematic media use (PMU; dark red) and left lingual is the overlapping cortical region of these constructs (green). For the most relevant regions of these two constructs see Fig. 4D as these are the ones overlapping with sensitivity to social feedback. (**B**) list of both cortical and subcortical brain regions predicting social anxiety. Some of these regions are integral in processing emotional and social stimuli and have been associated with social anxiety in the literature, such as amygdala, isthmus cingulate, orbitofrontal cortex. (**C**) regions related to problematic media use. Some of these regions play a role in emotional processing, reward processing and learning, but also habit formation, such as amygdala, lateral orbitofrontal cortex, nucleus accumbens and putamen, highlighting the potential neural underpinnings of problematic media use. All these brain regions performed better than the random benchmark consisting of values between 0 and 1 that are not relevant to predict any of the studied constructs.

Sample	Model	***k***	***n***	AIC
Adolescent	Null model	1	7718	1320008
	RL model	3	7718	1253746
	RL^d model	4	7718	1242755
Adult	Null model	1	8895	1566071
	RL model	3	8895	1460234
	RL^d model	4	8895	1442644
Both	Null model	1	16613	2886078
	RL model	3	16613	2713974
	RL^d model	4	16613	2685391

Table S1. Model performance using Akaike Information Criterion (AIC).

The table shows the sample, number of parameters (k), sample size (n) and AIC results for each model.

	**Adolescent**	**Adult**
**Likes (*M*)**	207.5	97.28
***τPost* (*M*)**	2.20	1.26
**Posts (*M*)**	71	132
**Followers**	1304	1856

Table S2. Descriptive statistics of Instagram trace data.

The table shows means (*M*) for likes, *τPost*, posts, and followers separately for adolescents and adults.

	Dependent variable:		
	Mood change T1	Mood change T2	Mood change T3
(Intercept)	1.85 (-6.96, 10.66)<br>p = 0.69	2.37 (-6.45, 11.20)<br>p = 0.60	4.22 (-6.91, 15.35)<br>p = 0.46
Social anxiety	0.79 (-2.31, 3.88)<br>p = 0.62	-1.95 (-5.05, 1.14)<br>p = 0.22	-1.17 (-5.07, 2.74)<br>p = 0.56
Age group	0.48 (-13.77, 14.74)<br>p = 0.95	1.49 (-12.78, 15.77)<br>p = 0.84	1.98 (-16.04, 19.99)<br>p = 0.84
Social anxiety X Age group	-1.31 (-5.91, 3.28)<br>p = 0.58	-1.21 (-5.81, 3.39)<br>p = 0.61	-2.52 (-8.33, 3.28)<br>p = 0.40
Observations	194	194	194

Table S3. The impact of social anxiety on mood change does not depend on the age group. Generalised linear models were fitted to individuals' mood change during the social media experiment to test the interaction of social anxiety and age group on mood change at each time point. Models report unstandardized coefficients. The 95% confidence intervals (CI) are in parenthesis and P values below the CI.

	Dependent variable:		
	Mood change T1	Mood change T2	Mood change T3
(Intercept)	15.47 (3.75, 27.19)<br>p = 0.02	-4.92 (-16.82, 6.97)<br>p = 0.42	10.55 (-4.47, 25.56)<br>p = 0.18
Problematic media use	-3.81 (-7.48, -0.13)<br>p = 0.05	0.73 (-3.00, 4.46)<br>p = 0.71	-3.08 (-7.78, 1.63)<br>p = 0.21
Age group	-22.44 (-43.16, -1.72)<br>p = 0.04	16.06 (-4.98, 37.09)<br>p = 0.14	-6.38 (-32.92, 20.17)<br>p = 0.64
Problematic media use X Age group	6.21 (-0.18, 12.61)<br>p = 0.06	-6.09 (-12.58, 0.40)<br>p = 0.07	0.12 (-8.07, 8.31)<br>p = 0.98
Observations	194	194	194

Table S4. The impact of problematic media use on mood change does not depend on the age group. Generalised linear models were fitted to individuals' mood change during the social media experiment to test the interaction of problematic media use and age group on mood change at each time point. Models report unstandardized coefficients. The 95% confidence intervals (CI) are in parenthesis and P values below the CI.

Adolescent

	Female (n=54)	Male (n=32)	Other (n=3)	Prefer not to say (n=4)
T1	*M* = 0.89<br>*SD* = 16	*M* = 0.47<br>*SD* = 24.4	*M* = 10.8<br>*SD* = 14.8	*M*= -21<br>*SD* =25.5
T2	*M* = -9.13<br>*SD* = 26.1	*M* =-1.25<br>*SD* =16.1	*M* = -3<br>*SD* = 10.1	*M* = -1.5<br>*SD* = 13.4
T3	*M* = -8.24<br>*SD* = 27.6	*M* = -0.78<br>*SD* = 26.8	M = 7.75<br>*SD* = 22.3	*M* = -22.5<br>*SD* = 12.0

Adult

	Female (n=35)	Male (n=67)
T1	*M* =1.03<br>*SD* = 12.0	*M* = 5.39<br>*SD* = 19.3
T2	*M* = -2.2<br>*SD* = 14.2	*M* = -2.97<br>*SD* =14.3
T3	*M* =-1.17<br>*SD* = 17.5	*M* = 2.42<br>*SD* = 20.3

Table S5. Descriptive statistics of mood change.

The table shows means (*M*) and standard deviations (*SD*) for mood changes across T1, T2, and T3 broken down by sexes and age groups.

	Dependent variable:	
	Mood	
	Teen	Adult
(Intercept)	0.89 (-5.38, 7.15)	1.03 (-4.63, 6.69)
	p = 0.79	p = 0.73
T2	-10.02 (-16.87, -3.17)	-3.23 (-9.62, 3.17)
	p = 0.005	p = 0.33
T3	-9.13 (-15.98, -2.28)	-2.20 (-8.59, 4.19)
	p = 0.01	p = 0.51
Sex	-0.42 (-10.69, 9.85)	4.36 (-2.62, 11.34)
	p = 0.94	p = 0.23
T2 X sex	8.30 (-2.93, 19.53)	-5.13 (-13.02, 2.76)
	p = 0.15	p = 0.21
T3 X sex	7.88 (-3.35, 19.11)	-0.77 (-8.66, 7.12)
	p = 0.17	p = 0.85

Table S6. Sex differences in mood changes in both adolescent and adult groups.

Linear mixed-effect models were fitted to individuals' mood changes during the social media experiment to test the effect of sex (reference: female) across all time points (reference: T1) during the social media experiment in the adolescent group and adult group. Models report unstandardized coefficients. The 95% confidence intervals (CI) are in parenthesis and P values are below the CI. We did not perform analysis with those identified as *other* or *prefer not to say* as the sample size was rather small.

	M
**Likes**	89
τPost	14.5
**Posts**	117
**Followers**	791

Table S7. Descriptive statistics of Instagram trace data.

The table shows means (*M*) for likes, *τPost*, posts, and followers of older youth.

Brain region	M	SD	p
**left amygdala**	0.0013525	0.0003994	***
**left putamen**	0.0043500	0.0007424	***
**left ventral DC**	0.0011329	0.0002475	***
**left fusiform**	0.0030620	0.0006971	***
**left precentral**	0.0023698	0.0003819	***
**left transverse temporal**	0.0052664	0.0010277	***
**right cuneus**	0.0017211	0.0004263	***
**right parahippocampal**	0.0082549	0.0019773	***
**right pars opercularis**	0.0025070	0.0004373	***
**right precentral**	0.0026064	0.0006724	***
**right superior parietal**	0.0011710	0.0002825	***
**right putamen**	0.0018896	0.0003649	***
**right ventral DC**	0.0025005	0.0004773	***

Table S8. Brain areas related to social feedback sensitivity.

Mean importance coefficient (*M*), standard deviations (SD) and *p* values (* $p < .05$; ** $p < .01$; *** $p < .001$) of the most important brain regions that beyond performing better than the random benchmark also showed statistical significance in sensitivity to social feedback.

Brain region	M	SD	p
**left amygdala**	0.0510504	0.0095503	***
**left cuneus**	0.0466151	0.0083780	***
**left inferior temporal**	0.0515859	0.0100246	***
**left isthmus cingulate**	0.0173571	0.0031780	***
**left lingual**	0.0796479	0.0146192	***
**left pericalcarine**	0.0550429	0.0109310	***
**left precentral**	0.0204938	0.0034631	***
**left superior parietal**	0.0576885	0.0107667	***
**right pars orbitalis**	0.0175300	0.0033611	***
**right ventral DC**	0.0162045	0.0038519	*

Table S9. Brain areas related to social anxiety.

Mean importance coefficient (M), standard deviations (SD) and p values (* $p < .05$; ** $p < .01$; *** $p < .001$) of the most important brain regions that beyond performing better than the random benchmark also showed statistical significance in predicting social anxiety.

Brain region	M	SD	p
**left amygdala**	0.0227703	0.0037461	***
**left lingual**	0.0071933	0.0014830	**
**left rostral anterior cingulate**	0.0072077	0.0013411	**
**left transverse temporal**	0.0241089	0.0040548	***
**right lateral orbitofrontal**	0.0502079	0.0085238	***
**right accumbens**	0.0103761	0.0019289	***
**right putamen**	0.0188564	0.0053830	***

Table S10. Brain areas related to problematic social media use.

Mean importance coefficient (*M*), standard deviations (SD) and *p* values (* $p < .05$; ** $p < .01$; *** $p < .001$) of the most important brain regions that beyond performing better than the random benchmark also showed statistical significance in predicting problematic social media use.

Screenshots of the experiment.

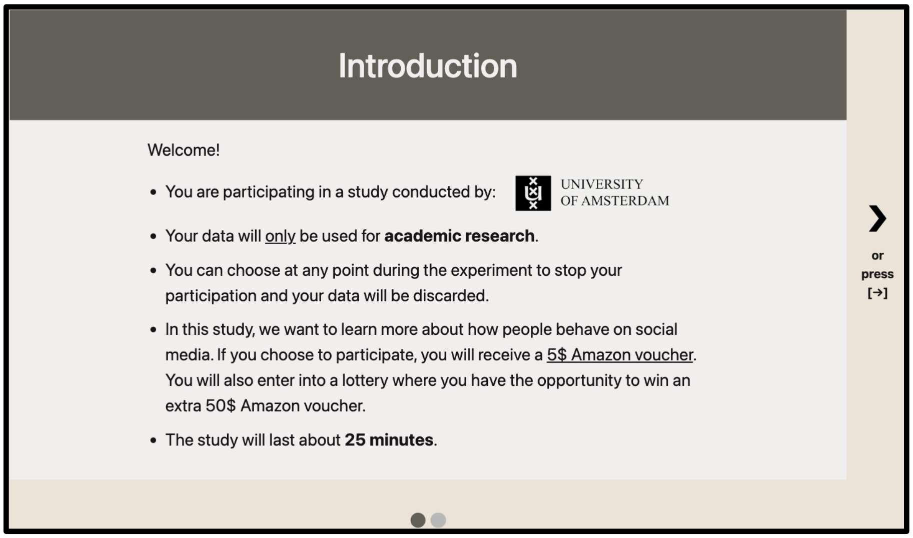

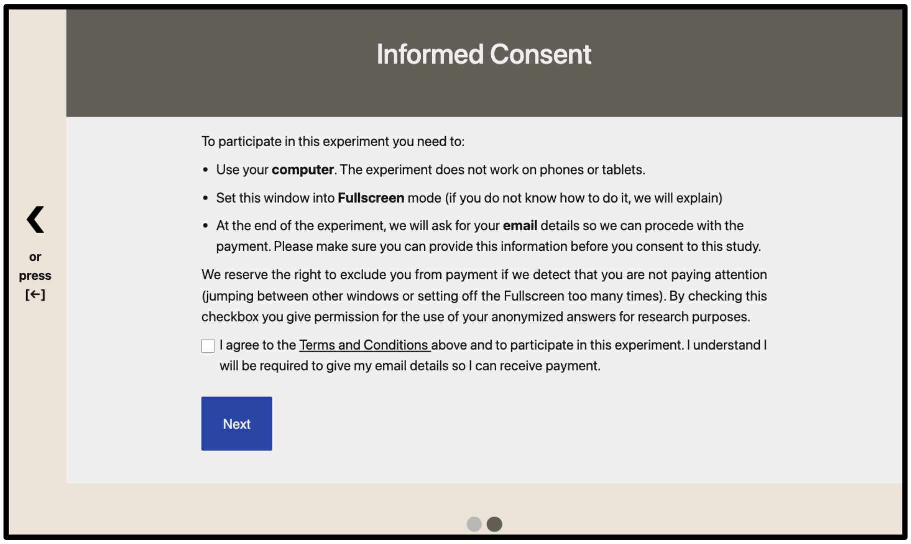

The Experiment

Please read carefully the information below. At the end of these instructions, you will be asked some comprehension questions.

- In this study, you will be using a platform that resembles aspects of social media (e.g. Instagram).
- You can either **post** "Memes" (humorous pictures) or **see and rate** Memes in an infinite Feed.
- Before the experiment begins, you will have have **two trials** to familiarize with the app. In the first trial, you will have 1 minute to try the infinite feed. In the second trial, you will be required to try post 4 times.
- After, you can post or see as many Memes you want for two sessions of **7 minutes** each.
- The figure shows a preview of the app. The left side displays the Feed. After you post for the first time, the information about your last post will be shown in the right side.

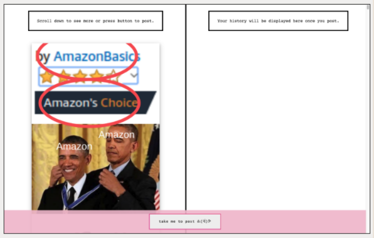

❯ or press [→]

Your Decisions

Each time you decide to **post** you can do so by selecting one Meme out of a set of 6 Memes.

You select a meme by pressing the button below the image.

The information about the posts and/or memes you like and dislike in the feed could be used in future academic research with peers of your age in an anonymized manner.

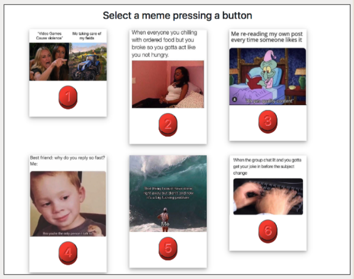

❮ or press [←]

❯ or press [→]

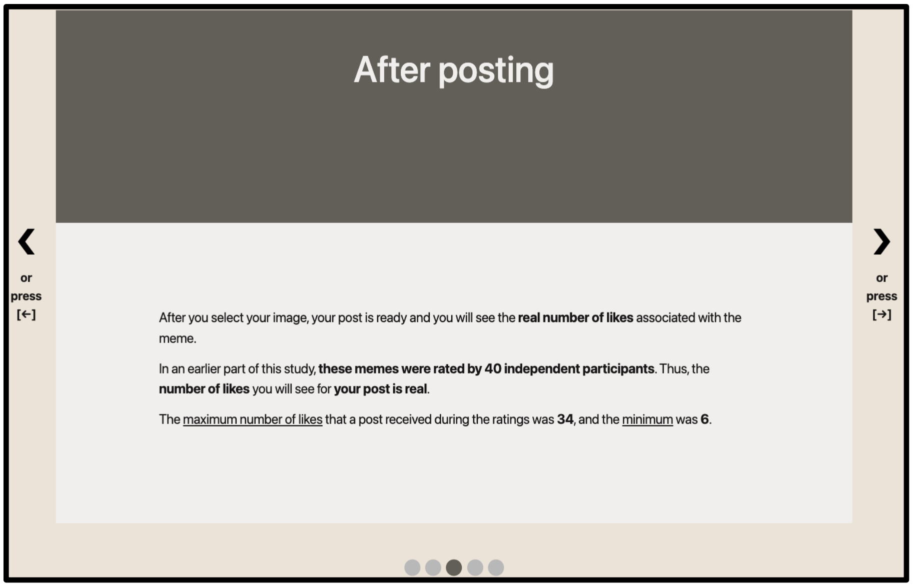
After posting
❮
or
press
[←]
❯
or
press
[→]
After you select your image, your post is ready and you will see the real number of likes associated with the meme.
In an earlier part of this study, these memes were rated by 40 independent participants. Thus, the number of likes you will see for your post is real.
The maximum number of likes that a post received during the ratings was 34, and the minimum was 6.

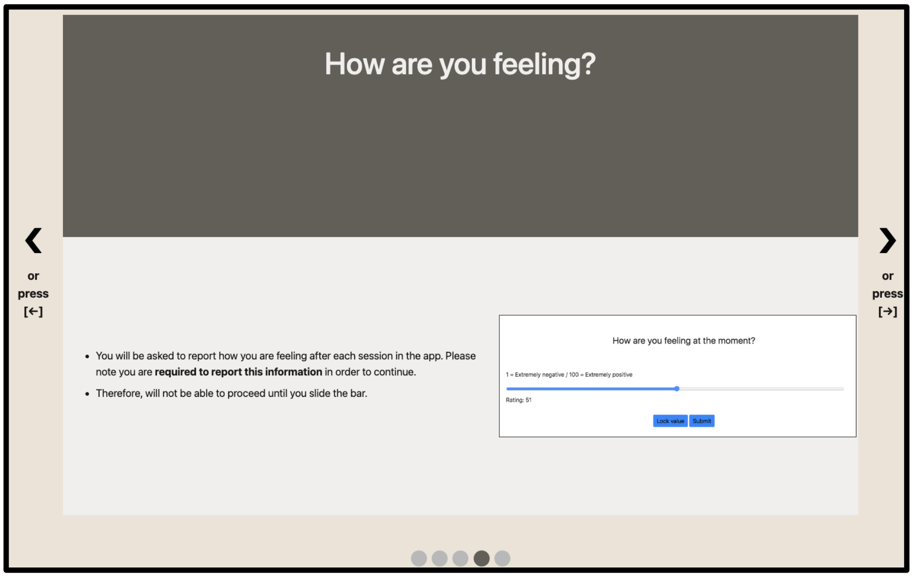
How are you feeling?
❮
or
press
[←]
❯
or
press
[→]
You will be asked to report how you are feeling after each session in the app. Please note you are required to report this information in order to continue.
Therefore, will not be able to proceed until you slide the bar.
How are you feeling at the moment?
1 = Extremely negative / 100 = Extremely positive
Rating: 51
Lock value
Submit

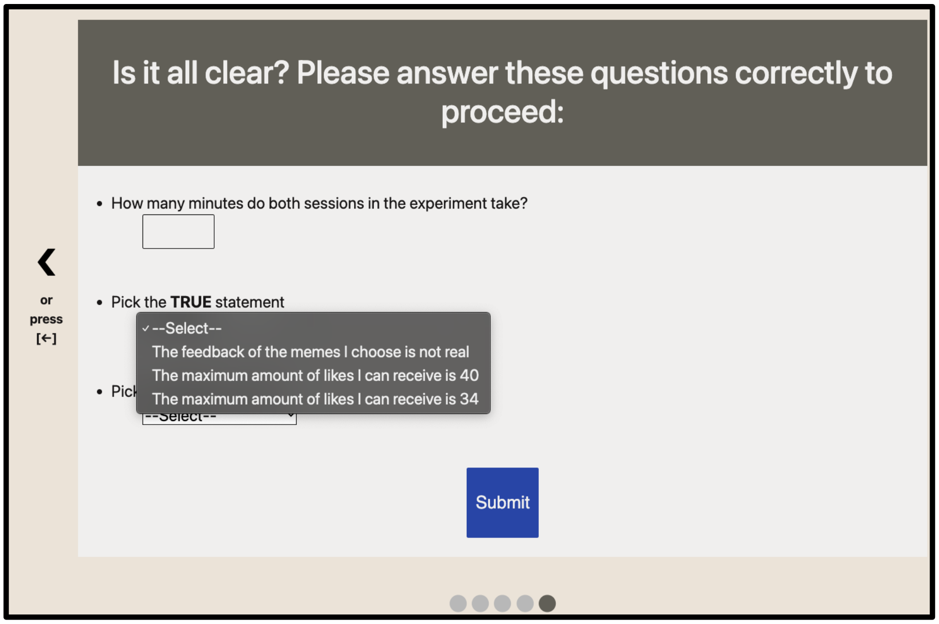

Is it all clear? Please answer these questions correctly to proceed:
How many minutes do both sessions in the experiment take?
Pick the TRUE statement
--Select--
The feedback of the memes I choose is not real
The maximum amount of likes I can receive is 40
The maximum amount of likes I can receive is 34
Pick
--Select--
Submit
or press [←]

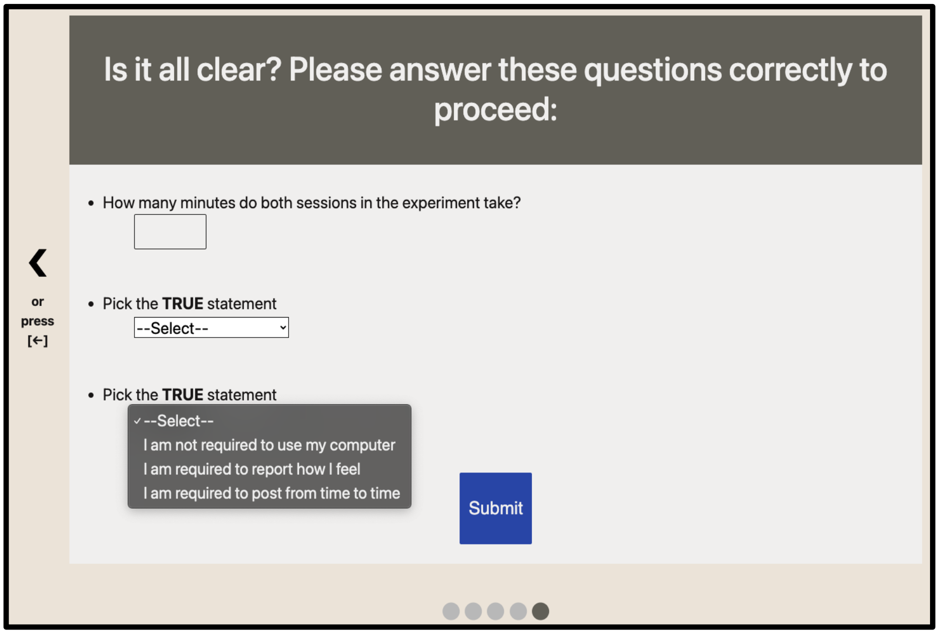

Is it all clear? Please answer these questions correctly to proceed:
How many minutes do both sessions in the experiment take?
Pick the TRUE statement
--Select--
Pick the TRUE statement
--Select--
I am not required to use my computer
I am required to report how I feel
I am required to post from time to time
Submit
or press [←]

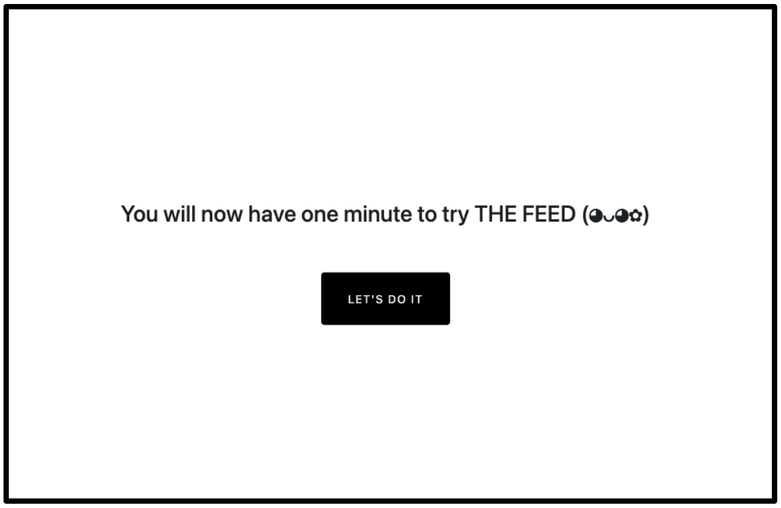

Participants could try the feed for a minute (see feed on main screen below).

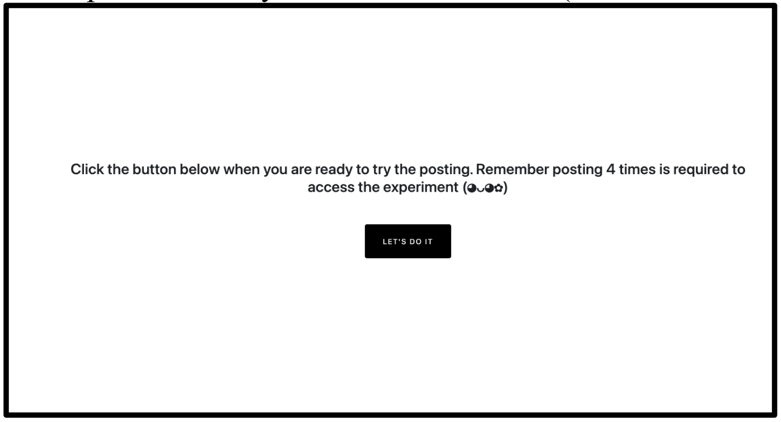

Trial in which participants could post and get familiar with the experiment (to post they would press the "Post" button on the main screen below).

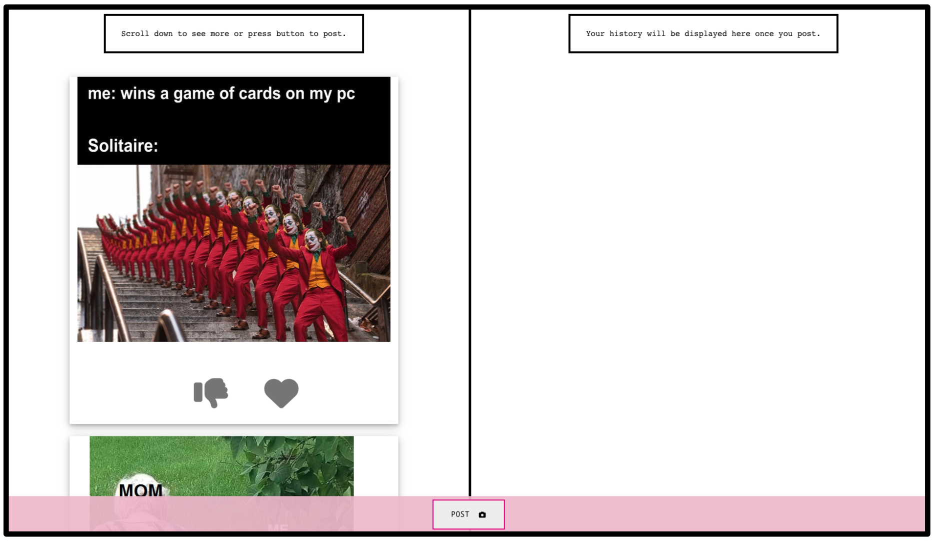

Main screen consists of both: the infinite feed on the left and the post history on the right. Participant did not yet post therefore the page is blank.

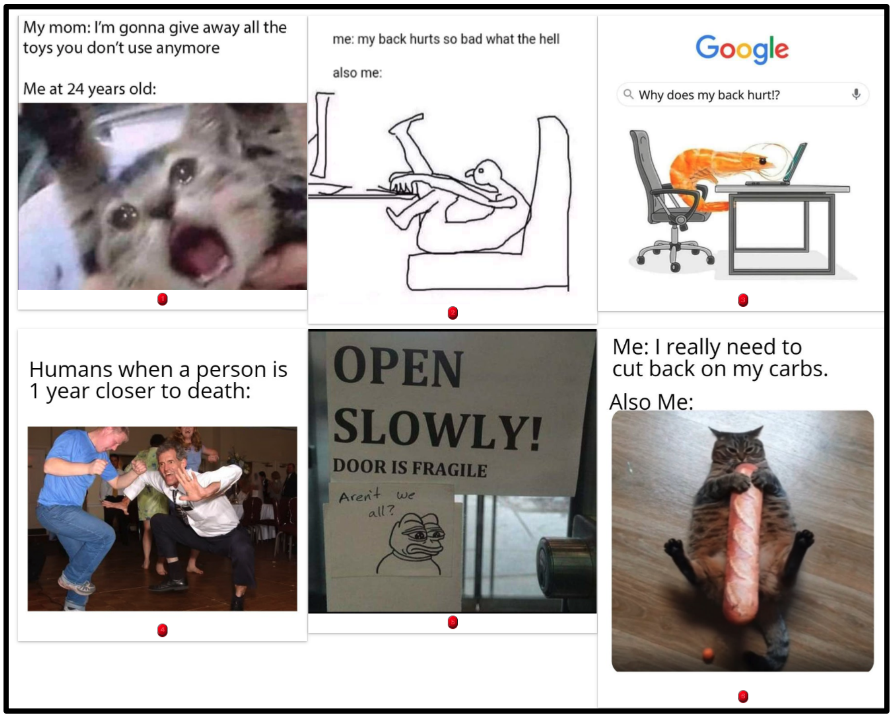

Set of 6 memes participants could select from.

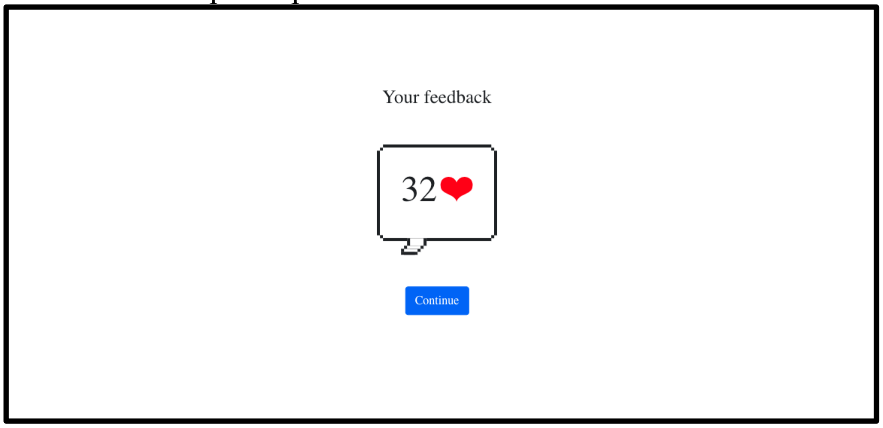

After posting, participants could see the feedback received for their post. This feedback is in the high reward condition.

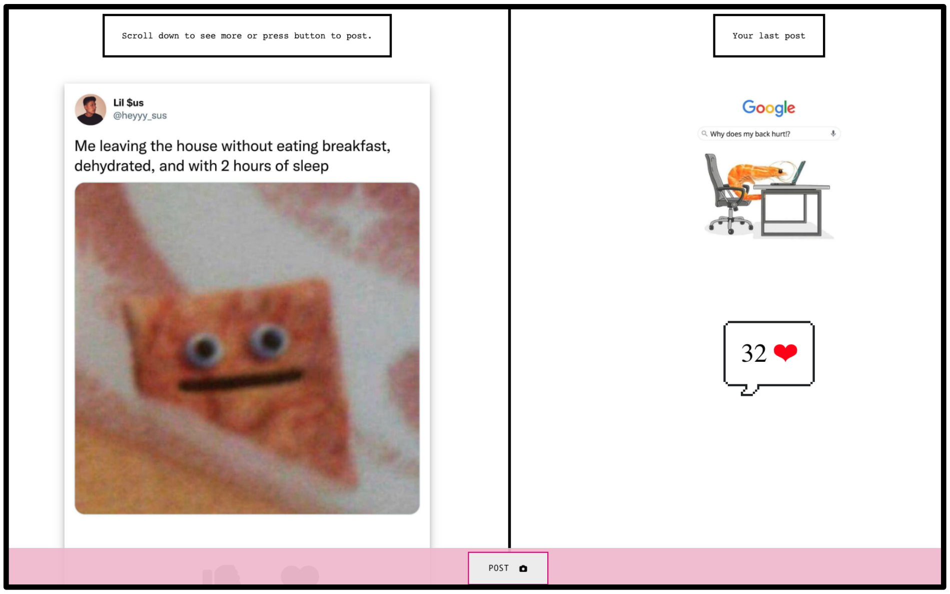

Back to the main screen, participants could continue scrolling or posting again. Now they can see their posting history consisting of the last post together with the feedback received. The same occurred in the low reward condition, except for the number of likes that was lower (between 6 and 18).

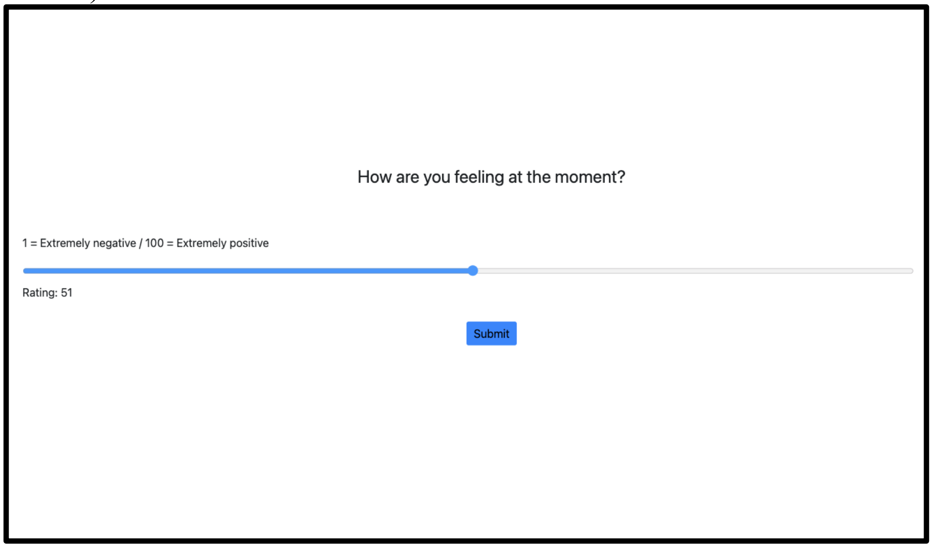

Measurement of mood which was done 3 times over the course of the experiment.

REFERENCES AND NOTES

1. UNESCO, *Youth* (UNESCO, 2024); www.unesco.org/en/youth.

2. R. E. Dahl, N. B. Allen, L. Wilbrecht, A. B. Suleiman, Importance of investing in adolescence from a developmental science perspective. *Nature* **554**, 441–450 (2018).

3. J. J. Arnett, Emerging adulthood: A theory of development from the late teens through the twenties. *Am. Psychol.* **55**, 469–480 (2000).

4. V. Bell, D. V. M. Bishop, A. K. Przybylski, The debate over digital technology and young people. *BMJ* **351**, h3064 (2015).

5. A. Orben, A. K. Przybylski, The association between adolescent well-being and digital technology use. *Nat. Hum. Behav.* **3**, 173–182 (2019).

6. M. T. Maza, K. A. Fox, S.-J. Kwon, J. E. Flannery, K. A. Lindquist, M. J. Prinstein, E. H. Telzer, Association of habitual checking behaviors on social media with longitudinal functional brain development. *JAMA Pediatr.* **177**, 160–167 (2023).

7. B. Keles, N. McCrae, A. Grealish, A systematic review: The influence of social media on depression, anxiety and psychological distress in adolescents. *Int. J. Adolesc. Youth* **25**, 79–93 (2020).

8. N. McCrae, S. Gettings, E. Purssell, Social media and depressive symptoms in childhood and adolescence: A systematic review. *Adolesc. Res. Rev.* **2**, 315–330 (2017).

9. M. Achterberg, A. Becht, R. van der Cruijsen, I. H. van de Groep, J. P. Spaans, E. Klapwijk, E. A. Crone, Longitudinal associations between social media use, mental well-being and structural brain development across adolescence. *Dev. Cogn. Neurosci.* **54**, 101088 (2022).

10. S. L. Garrett, K. Burnell, E. L. Armstrong-Carter, M. J. Prinstein, E. H. Telzer, Linking video chatting, phone calling, text messaging, and social media with peers to adolescent connectedness. *J. Res. Adolesc.* **33**, 1222–1234 (2023).

11. A. M. Rosenthal-von der Pütten, M. R. Hastall, S. Köcher, C. Meske, T. Heinrich, F. Labrenz, S. Ocklenburg, "Likes" as social rewards: Their role in online social comparison and decisions to like other People's selfies. *Comput. Human Behav.* **92**, 76–86 (2019).

12. C. G. Davey, N. B. Allen, B. J. Harrison, D. B. Dwyer, M. Yücel, Being liked activates primary reward and midline self-related brain regions. *Hum. Brain Mapp.* **31**, 660–668 (2010).

13. A. L. Burrow, N. Rainone, How many likes did I get?: Purpose moderates links between positive social media feedback and self-esteem. *J. Exp. Soc. Psychol.* **69**, 232–236 (2017).

14. D. Smith, T. Leonis, S. Anandavalli, Belonging and loneliness in cyberspace: Impacts of social media on adolescents' well-being. *Aust. J. Psychol.* **73**, 12–23 (2021).

15. B. Lindström, M. Bellander, D. T. Schultner, A. Chang, P. N. Tobler, D. M. Amodio, A computational reward learning account of social media engagement. *Nat. Commun.* **12**, 1311 (2021).

16. A. L. Zell, L. Moeller, Are you happy for me … on Facebook? The potential importance of "likes" and comments. *Comput. Human Behav.* **78**, 26–33 (2018).

17. J. P. Jamieson, S. G. Harkins, K. D. Williams, Need threat can motivate performance after ostracism. *Pers. Soc. Psychol. Bull.* **36**, 690–702 (2010).

18. A. Irmer, F. Schmiedek, Associations between youth's daily social media use and well-being are mediated by upward comparisons. *Commun. Psychol.* **1**, 12 (2023).

19. E. P. Shulman, A. R. Smith, K. Silva, G. Icenogle, N. Duell, J. Chein, L. Steinberg, The dual systems model: Review, reappraisal, and reaffirmation. *Dev. Cogn. Neurosci.* **17**, 103–117 (2016).

20. M. E. Quarmley, B. D. Nelson, T. Clarkson, L. K. White, J. M. Jarcho, I knew you weren't going to like me! Neural response to accurately predicting rejection is associated with anxiety and depression. *Front. Behav. Neurosci.* **13**, 219 (2019).

21. A. C. K. van Duijvenvoorde, S. Peters, B. R. Braams, E. A. Crone, What motivates adolescents? Neural responses to rewards and their influence on adolescents' risk taking, learning, and cognitive control. *Neurosci. Biobehav. Rev.* **70**, 135–147 (2016).

22. A. Galván, The teenage brain: Sensitivity to rewards. *Curr. Dir. Psychol. Sci.* **22**, 88–93 (2013).

23. J. S. Silk, G. J. Siegle, K. H. Lee, E. E. Nelson, L. R. Stroud, R. E. Dahl, Increased neural response to peer rejection associated with adolescent depression and pubertal development. *Soc. Cogn. Affect. Neurosci.* **9**, 1798–1807 (2014).

24. M. J. Prinstein, J. W. Aikins, Cognitive moderators of the longitudinal association between peer rejection and adolescent depressive symptoms. *J. Abnorm. Child Psychol.* **32**, 147–158 (2004).

25. C. L. Odgers, M. R. Jensen, Annual research review: Adolescent mental health in the digital age: Facts, fears, and future directions. *J. Child Psychol. Psychiatry* **61**, 336–348 (2020).

26. M. Panayiotou, L. Black, P. Carmichael-Murphy, P. Qualter, N. Humphrey, Time spent on social media among the least influential factors in adolescent mental health: Preliminary results from a panel network analysis. *Nat. Ment. Health* **1**, 316–326 (2023).

27. D. A. Parry, B. I. Davidson, C. J. R. Sewall, J. T. Fisher, H. Mieczkowski, D. S. Quintana, A systematic review and meta-analysis of discrepancies between logged and self-reported digital media use. *Nat. Hum. Behav.* **5**, 1535–1547 (2021).

28. M. Sultan, C. Scholz, W. van den Bos, Leaving traces behind: Using social media digital trace data to study adolescent wellbeing. *Comput. Hum. Behav. Rep.* **10**, 100281 (2023).

29. L. N. Hodes, K. G. F. Thomas, Smartphone screen time: Inaccuracy of self-reports and influence of psychological and contextual factors. *Comput. Human Behav.* **115**, 106616 (2021).

30. A. J. Fuligni, A. Galván, Young people need experiences that boost their mental health. *Nature* **610**, 253–256 (2022).

31. UNICEF, *Adolescent Mental Health Statistics* (UNICEF DATA, 2019); https://data.unicef.org/topic/child-health/mental-health/.

32. R. J. Herrnstein, On the law of effect. *J. Exp. Anal. Behav.* **13**, 243–266 (1970).

33. Y. Niv, N. D. Daw, D. Joel, P. Dayan, Tonic dopamine: Opportunity costs and the control of response vigor. *Psychopharmacology* **191**, 507–520 (2007).

34. E. A. Crone, E. A. Konijn, Media use and brain development during adolescence. *Nat. Commun.* **9**, 588 (2018).

35. K. Han, S. Lee, J. Y. Jang, Y. Jung, D. Lee, Teens are from mars, adults are from venus: Analyzing and predicting age groups with behavioral characteristics in instagram, in *Proceedings of the 8th ACM Conference on Web Science, WebSci '16* (Association for Computing Machinery, 2016), pp. 35–44; https://doi.org/10.1145/2908131.2908160.

36. R. B. Rutledge, N. Skandali, P. Dayan, R. J. Dolan, A computational and neural model of momentary subjective well-being. *Proc. Natl. Acad. Sci. U.S.A.* **111**, 12252–12257 (2014).

37. E. Eldar, Y. Niv, Interaction between emotional state and learning underlies mood instability. *Nat. Commun.* **6**, 6149 (2015).

38. R. C. Bowen, Y. Wang, L. Balbuena, A. Houmphan, M. Baetz, The relationship between mood instability and depression: Implications for studying and treating depression. *Med. Hypotheses* **81**, 459–462 (2013).

39. S. Reich, F. M. Schneider, L. Heling, Zero likes—Symbolic interactions and need satisfaction online. *Comput. Human Behav.* **80**, 97–102 (2018).

40. B. Taber-Thomas, K. Pérez-Edgar, Eds., in *Emerging Adulthood Brain Development* (Oxford Univ. Press, 2014), chap. 8.

41. L. E. Sherman, A. A. Payton, L. M. Hernandez, P. M. Greenfield, M. Dapretto, The power of the like in adolescence: Effects of peer influence on neural and behavioral responses to social media. *Psychol. Sci.* **27**, 1027–1035 (2016).

42. J. Nesi, M. J. Prinstein, In search of likes: Longitudinal associations between adolescents' digital status seeking and health-risk behaviors. *J. Clin. Child Adolesc. Psychol.* **48**, 740–748 (2019).

43. E. A. Crone, M. Achterberg, S. Dobbelaar, S. Euser, B. van den Bulk, M. van der Meulen, L. van Drunen, L. M. Wierenga, M. J. Bakermans-Kranenburg, Neural and behavioral signatures of social evaluation and adaptation in childhood and adolescence: The Leiden consortium on individual development (L-CID). *Dev. Cogn. Neurosci.* **45**, 100805 (2020).

44. E. Towner, G. Chierchia, S.-J. Blakemore, Sensitivity and specificity in affective and social learning in adolescence. *Trends Cogn. Sci.* **27**, 642–655 (2023).

45. C. Sebastian, E. Viding, K. D. Williams, S.-J. Blakemore, Social brain development and the affective consequences of ostracism in adolescence. *Brain Cogn.* **72**, 134–145 (2010).

46. S. J. Blakemore, K. L. Mills, Is adolescence a sensitive period for sociocultural processing? *Annu. Rev. Psychol.* **65**, 187–207 (2014).

47. A. R. Smith, L. Steinberg, N. Strang, J. Chein, Age differences in the impact of peers on adolescents' and adults' neural response to reward. *Dev. Cogn. Neurosci.* **11**, 75–82 (2015).

48. L. H. Somerville, Special issue on the teenage brain: Sensitivity to social evaluation. *Curr. Dir. Psychol. Sci.* **22**, 121–127 (2013).

49. A. E. Guyer, J. S. Silk, E. E. Nelson, The neurobiology of the emotional adolescent: From the inside out. *Neurosci. Biobehav. Rev.* **70**, 74–85 (2016).

50. S. H. Sperry, M. A. Walsh, T. R. Kwapil, Emotion dynamics concurrently and prospectively predict mood psychopathology. *J. Affect. Disord.* **261**, 67–75 (2020).

51. D. F. Maciejewski, L. Keijsers, P. A. C. van Lier, S. J. T. Branje, W. H. J. Meeus, H. M. Koot, Most fare well—But some do not: Distinct profiles of mood variability development and their association with adjustment during adolescence. *Dev. Psychol.* **55**, 434–448 (2019).

52. M. Anderson, J. Jang, *Teens, Social Media & Technology 2018* (Pew Research Center, 2018); www.pewresearch.org/internet/2018/05/31/teens-social-media-technology-2018/.

53. A. Orben, T. Dienlin, A. K. Przybylski, Social media's enduring effect on adolescent life satisfaction. *Proc. Natl. Acad. Sci. U.S.A.* **116**, 10226–10228 (2019).

54. J. Nesi, M. J. Prinstein, Using social media for social comparison and feedback-seeking: Gender and popularity moderate associations with depressive symptoms. *J. Abnorm. Child Psychol.* **43**, 1427–1438 (2015).

55. O. Bartra, J. T. McGuire, J. W. Kable, The valuation system: A coordinate-based meta-analysis of BOLD fMRI experiments examining neural correlates of subjective value. *Neuroimage* **76**, 412–427 (2013).

56. N. I. Eisenberger, M. D. Lieberman, K. D. Williams, Does rejection hurt? An FMRI study of social exclusion. *Science* **302**, 290–292 (2003).

57. D. S. Chester, D. R. Lynam, R. Milich, C. N. DeWall, Neural mechanisms of the rejection-aggression link. *Soc. Cogn. Affect. Neurosci.* **13**, 501–512 (2018).

58. P. Wikman, M. Moisala, A. Ylinen, J. Lindblom, S. Leikas, K. Salmela-Aro, K. Lonka, B. Güroğlu, K. Alho, Brain Responses to peer feedback in social media are modulated by valence in late adolescence. *Front. Behav. Neurosci.* **16**, 790478 (2022).

59. A. E. Guyer, V. R. Choate, D. S. Pine, E. E. Nelson, Neural circuitry underlying affective response to peer feedback in adolescence. *Soc. Cogn. Affect. Neurosci.* **7**, 81–92 (2012).

60. B. Gunther Moor, L. van Leijenhorst, S. A. R. B. Rombouts, E. A. Crone, M. W. Van der Molen, Do you like me? Neural correlates of social evaluation and developmental trajectories. *Soc. Neurosci.* **5**, 461–482 (2010).

61. S. Rathje, J. J. Van Bavel, S. van der Linden, Out-group animosity drives engagement on social media. *Proc. Natl. Acad. Sci. U.S.A.* **118**, e2024292118 (2021).

62. J. L. Pouwels, T. Araujo, W. van Atteveldt, M. Bachl, P. M. Valkenburg, Integrating communication science and computational methods to study content-based social media effects. *Commun. Methods Meas.* **18**, 115–123 (2023).

63. I. Beyens, J. L. Pouwels, I. I. van Driel, L. Keijsers, P. M. Valkenburg, Social media use and adolescents' well-being: Developing a typology of person-specific effect patterns. *Communic. Res.* **51**, 691–716 (2021).

64. E. A. Crone, R. E. Dahl, Understanding adolescence as a period of social-affective engagement and goal flexibility. *Nat. Rev. Neurosci.* **13**, 636–650 (2012).

65. C. de Vreese, D. A. de Vries, J. Piotrowski, *Digital Competence Across the Lifespan* (Center for Open Science, 2021); https://osf.io/d5c7n/.

66. F. Laaber, A. Florack, T. Koch, M. Hubert, Digital maturity: Development and validation of the Digital Maturity Inventory (DIMI). *Comput. Human Behav.* **143**, 107709 (2023).

67. P. Green, C. J. MacLeod, SIMR: An R package for power analysis of generalized linear mixed models by simulation. *Methods Ecol. Evol.* **7**, 493–498 (2016).

68. M. Brooks, K. Kristensen, K. van Benthem, A. Magnusson, C. Berg, A. Nielsen, H. Skaug, M. Mächler, B. Bolker, GlmmTMB balances speed and flexibility among packages for zero-inflated generalized linear mixed modeling. *R J.* **9**, 378–400 (2017).

69. N. J. Tustison, B. B. Avants, P. A. Cook, Y. Zheng, A. Egan, P. A. Yushkevich, J. C. Gee, N4ITK: Improved N3 bias correction. *IEEE Trans. Med. Imaging* **29**, 1310–1320 (2010).

70. B. B. Avants, C. L. Epstein, M. Grossman, J. C. Gee, Symmetric diffeomorphic image registration with cross-correlation: Evaluating automated labeling of elderly and neurodegenerative brain. *Med. Image Anal.* **12**, 26–41 (2008).

71. Y. Zhang, M. Brady, S. Smith, Segmentation of brain MR images through a hidden Markov random field model and the expectation-maximization algorithm. *IEEE Trans. Med. Imaging* **20**, 45–57 (2001).

72. V. Fonov, A. C. Evans, K. Botteron, C. R. Almli, R. C. McKinstry, D. L. Collins; Brain Development Cooperative Group, Unbiased average age-appropriate atlases for pediatric studies. *Neuroimage* **54**, 313–327 (2011).

73. R. S. Desikan, F. Ségonne, B. Fischl, B. T. Quinn, B. C. Dickerson, D. Blacker, R. L. Buckner, A. M. Dale, R. P. Maguire, B. T. Hyman, M. S. Albert, R. J. Killiany, An automated labeling system for subdividing the human cerebral cortex on MRI scans into gyral based regions of interest. *Neuroimage* **31**, 968–980 (2006).

74. B. Fischl, D. H. Salat, E. Busa, M. Albert, M. Dieterich, C. Haselgrove, A. van der Kouwe, R. Killiany, D. Kennedy, S. Klaveness, A. Montillo, N. Makris, B. Rosen, A. M. Dale, Whole brain segmentation: Automated labeling of neuroanatomical structures in the human brain. *Neuron* **33**, 341–355 (2002).

75. F. Pedregosa, G. Varoquaux, A. Gramfort, V. Michel, B. Thirion, O. Grisel, M. Blondel, P. Prettenhofer, R. Weiss, V. Dubourg, J. Vanderplas, A. Passos, D. Cournapeau, M. Brucher, M. Perrot, É. Duchesnay, Scikit-learn: Machine learning in Python. *J. Mach. Learn. Res.* **12**, 2825–2830 (2011).

76. A. Altmann, L. Toloşi, O. Sander, T. Lengauer, Permutation importance: A corrected feature importance measure. *Bioinformatics* **26**, 1340–1347 (2010).

77. G.-J. Meerkerk, R. J. J. M. Van Den Eijnden, A. A. Vermulst, H. F. L. Garretsen, The Compulsive Internet Use Scale (CIUS): Some psychometric properties. *Cyberpsychol. Behav.* **12**, 1–6 (2009).

78. A. L. Nichols, G. D. Webster, Designing a brief measure of social anxiety: Psychometric support for a three-item version of the Interaction Anxiousness Scale (IAS-3). *Pers. Individ. Dif.* **79**, 110–115 (2015).

79. RStudio Team, *Integrated Development for R* (Rstudios, Inc., 2020).

80. H. Akaike, Information theory and an extension of the maximum likelihood principle, in *Selected Papers of Hirotugu Akaike*, E. Parzen, K. Tanabe, G. Kitagawa, Eds. (Springer, 1998), pp. 199–213.

81. L. G. Weil, S. M. Fleming, I. Dumontheil, E. J. Kilford, R. S. Weil, G. Rees, R. J. Dolan, S.-J. Blakemore, The development of metacognitive ability in adolescence. *Conscious. Cogn.* **22**, 264–271 (2013).

82. M. Paulus, N. Tsalas, J. Proust, B. Sodian, Metacognitive monitoring of oneself and others: Developmental changes during childhood and adolescence. *J. Exp. Child Psychol.* **122**, 153–165 (2014).

83. S. C. Sung, E. Porter, D. J. Robinaugh, E. H. Marks, L. M. Marques, M. W. Otto, M. H. Pollack, N. M. Simon, Mood regulation and quality of life in social anxiety disorder: An examination of generalized expectancies for negative mood regulation. *J. Anxiety Disord.* **26**, 435–441 (2012).

84. P. Peng, Y. Liao, Six addiction components of problematic social media use in relation to depression, anxiety, and stress symptoms: A latent profile analysis and network analysis. *BMC Psychiatry* **23**, 321 (2023).

85. D. F. Maciejewski, P. A. C. van Lier, S. J. T. Branje, W. H. J. Meeus, H. M. Koot, A 5-year longitudinal study on mood variability across adolescence using daily diaries. *Child Dev.* **86**, 1908–1921 (2015).

86. T. Mahalingham, P. M. McEvoy, P. J. F. Clarke, Assessing the validity of self-report social media use: Evidence of No relationship with objective smartphone use. *Comput. Human Behav.* **140**, 107567 (2023).

87. S. P. Ahmed, B. Piera Pi-Sunyer, M. E. Moses-Payne, A.-L. Goddings, L. G. Speyer, W. Kuyken, T. Dalgleish, S.-J. Blakemore, The role of self-referential and social processing in the

relationship between pubertal status and difficulties in mental health and emotion regulation in adolescent girls in the UK. *Dev. Sci.* **27** e13503 (2024).

88. J. M. Twenge, G. N. Martin, Gender differences in associations between digital media use and psychological well-being: Evidence from three large datasets. *J. Adolesc.* **79**, 91–102 (2020).

89. S. Champely, C. Ekstrom, P. Dalgaard, J. Gill, S. Weibelzahl, A. Anandkumar, C. Ford, R. Volcic, H. De Rosario, *pwr: Basic Functions for Power Analysis* (2017); R package version 1.2-2.
